# Does Water Enable Porosity in Aluminosilicate Zeolites? Porous Frameworks versus Dense Minerals

**DOI:** 10.1021/acs.cgd.2c01476

**Published:** 2023-03-24

**Authors:** Karel Asselman, Mohamed Haouas, Maarten Houlleberghs, Sambhu Radhakrishnan, Wauter Wangermez, Christine E. A. Kirschhock, Eric Breynaert

**Affiliations:** † Centre for Surface Chemistry and Catalysis-Characterisation and Application Team (COK-KAT), KU Leuven, Leuven 3001, Belgium; ‡ Institut Lavoisier de Versailles, Université Paris-Saclay, UVSQ, CNRS, 78000 Versailles, France; § NMRCoRe-NMR-X-Ray platform for Convergence Research, KU Leuven, Leuven 3001, Belgium

## Abstract

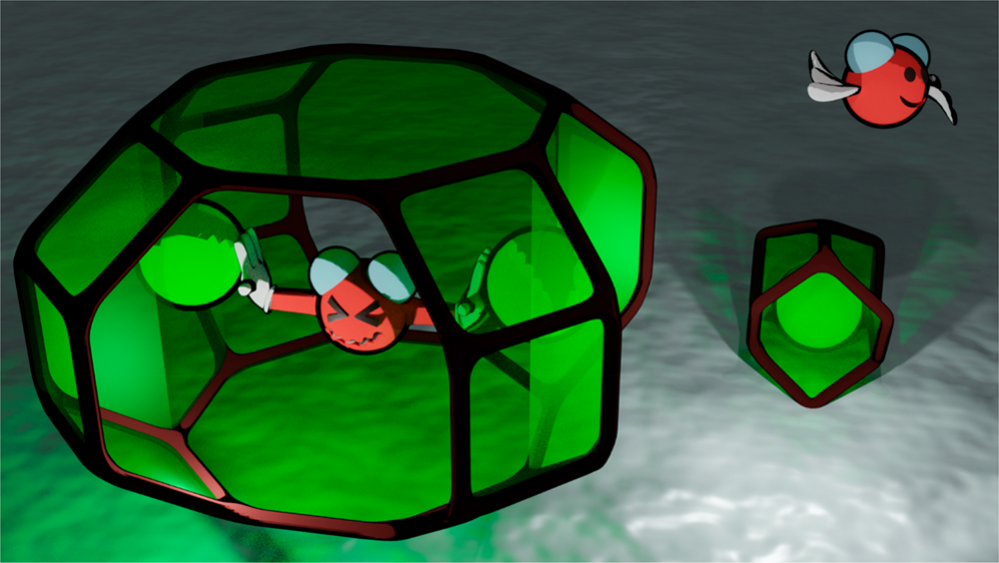

Recently identified zeolite precursors consisting of concentrated, hyposolvated homogeneous alkalisilicate liquids, hydrated silicate ionic liquids (HSIL), minimize correlation of synthesis variables and enable one to isolate and examine the impact of complex parameters such as water content on zeolite crystallization. HSIL are highly concentrated, homogeneous liquids containing water as a reactant rather than bulk solvent. This simplifies elucidation of the role of water during zeolite synthesis. Hydrothermal treatment at 170 °C of Al-doped potassium HSIL with chemical composition 0.5SiO_2_:1KOH:*x*H_2_O:0.013Al_2_O_3_ yields porous merlinoite (MER) zeolite when H_2_O/KOH exceeds 4 and dense, anhydrous megakalsilite when H_2_O/KOH is lower. Solid phase products and precursor liquids were fully characterized using XRD, SEM, NMR, TGA, and ICP analysis. Phase selectivity is discussed in terms of cation hydration as the mechanism, allowing a spatial cation arrangement enabling the formation of pores. Under water deficient conditions, the entropic penalty of cation hydration in the solid is large and cations need to be entirely coordinated by framework oxygens, leading to dense, anhydrous networks. Hence, the water activity in the synthesis medium and the affinity of a cation to either coordinate to water or to aluminosilicate decides whether a porous, hydrated, or a dense, anhydrous framework is formed.

## Abstract 2

In highly concentrated alkaline aluminosilicate synthesis solutions, varying the water content selects between non-porous phases and hydrated zeolites. Favorable enthalpy and unfavorable entropy of confined hydration water compete in determining the global free energy of the crystallization system.

## 1 Introduction

Zeolites belong to the tectosilicates, and their structural and chemical properties are of high relevance for numerous applications in industry. Their synthesis is commonly achieved by hydrothermal treatment of gel-phases, mimicking zeolite formation in geochemical processes.^1^ While full control of the synthesis route and understanding of the nucleation and crystal growth mechanisms are still an active field of research,^2−4^ the influence of reactants and reaction variables has extensively been studied and general trends start to emerge.^5−7^ Hydrothermal zeolite synthesis comprises a vast and strongly correlated parameter space, with a myriad of variables impacting crystallization and phase behavior. This includes synthesis time and temperature, molar composition, the source of Si and Al, etc. Among these compositional descriptors, the influence of the cation type is the most documented. Under identical conditions, different (earth-) alkali metal ions select for distinct zeolite topologies due to their preference for different coordination sites within the pore structures.^8−10^ The alkalinity, or [OH^–^] content of the precursor mixture, impacts not only the interparticle repulsion and aggregation processes, but also the (de)oligomerization and equilibrium distribution of soluble (alumino)silicate precursors,^11−13^ governing the final framework Si/Al and corresponding topology.^9,14^ Of all compositional descriptors, the impact of water content on the liquid chemistry and resulting synthesis product is understood the least. In early publications on zeolite synthesis, dating back to the 1940s, the water content was often not reported as it was regarded as a solvent rather than a reactant. Today, water content is acknowledged as a critical synthesis variable in zeolite synthesis. It has been shown to impact both gelation and crystallization kinetics,^15−17^ and to influence the final aluminum content as well as the framework topology.^14^ With increasing water content, phase transitions from Na-SOD to Na-LTA,^18,19^ K-EDI to K-CHA,^14^ and Na-MOR to Na-MFI,^20^ have been reported, with certain topologies requiring more dilute synthesis systems. For siliceous zeolite Beta polymorphs (BEA, BEB, BEC), polymorph selection is theoretically predicted to be affected by the water content. The study ascribes water to exert a stabilizing influence on void space not occupied by the organic structure-directing agent.^21^ For synthesis at moderate temperatures (<100 °C), excess water has been shown to potentially be detrimental for crystallization. Lowering the overall supersaturation of framework forming solutes^16^ and weakening the association strength between alkali cation and aluminosilicate oligomers in solution through excess cation hydration, can indeed prevent crystallization.^22^ Structural water stabilizes porous frameworks via favorable hydration enthalpies with extra framework cations, and pore-filling effects.^23^ Such energetic contribution becomes more and more relevant with increasing framework aluminum, corresponding to increasing hydrophilicity and cation content.^24^

Formation of hydrated/porous or anhydrous/dense aluminosilicate phases or feldspars also depends on the alkali cation. Under typical hydrothermal synthesis conditions (100–180 °C), and in the absence of other structure-directing agents, Cs^+^ nearly exclusively selects for anhydrous phases such as ANA (synthetic pollucite) and ABW, while Na^+^ almost always yields hydrated zeolite phases. In this contribution, we demonstrate K^+^, exhibiting an intermediate affinity for water, to yield both hydrated zeolites or anhydrous aluminosilicate tectosilicates using slight changes in the water content of the synthesis as a phase selector. This observation prompted the current detailed investigation of the impact of water content on zeolite synthesis in concentrated synthesis media, revealing its impact on the formation of potassium aluminosilicate ion pairs and their impact on phase selection, resulting in either hydrated or dense phases.

Water content is a complex synthesis variable, highly correlated to many other synthesis descriptors. It impacts the charge density and pH of the liquid and influences cation coordination behavior and its interaction with framework-building species, and it changes the concentration and chemical potential of all other solutes. Therefore, investigating the role of water in zeolite synthesis benefits from the use of a model crystallization system, allowing isolation of this descriptor and complete characterization of its impact on liquid chemistry as well as final crystal products.

A promising case is zeolite synthesis using hydrated silicate ionic liquids (HSIL).^2,14,25^ Owing to high ionicity and low water and aluminum content in the presence of alkali cations, gel formation can be prevented. These clear, homogeneous precursor liquids allow detailed characterization using nuclear magnetic resonance (NMR) spectroscopy, dynamic light scattering, and electrochemical impedance spectroscopy.^25,26^ In addition, their monophasic, homogeneous nature, with rapid equilibration of the molecular species^2^ and absence of gel and colloidal phases, allows systematic exploration of individual synthesis variables. Native siliceous HSIL media, prior to aluminate addition, appear to be in chemical equilibrium. No changes have been detected at periods of more than one year, and the system is presumed to be indefinitely stable. This changes as soon as aluminate is introduced. At substantial aluminate concentrations, aggregation and colloid or gel formation occurs instantaneous. However, keeping the aluminate concentration sufficiently low, the aluminate fully dissolves, forming small, dynamic aluminosilicate oligomers, as indicated by DLS and NMR characterization of the synthesis solutions demonstrating the absence of larger particulates.^22,27^ The critical aluminate content is typically very low, but depends strongly on the batch stoichiometry. This was highlighted in a previous study, where the demarcation between compositions yielding homogeneous, colloid-free liquids, and colloidal systems was identified.^14^ Batch compositions in the 0.5SiO_2_–0.013Al_2_O_3_–*y*MOH–*x*H_2_O system yielded homogeneous, particulate-free liquids when both *y* > 1 and *x*/*y* < 15, i.e., requiring sufficient alkalinity and also limited water content. For precursor mixtures outside this compositional range, the aluminate content must be lowered even further. However, any precursor liquid, even the homogeneous liquids where aluminate is initially in a fully dissolved state, is metastable upon introduction of aluminate and will evolve over time. Depending on the starting stoichiometry, the precursor will eventually crystallize zeolites or evolve into an amorphous aluminosilicate phase, even at room temperature. We indeed observed macroscopic changes at room temperature in the time frame of several days to months, depending on the batch composition and type of alkali cation. Under hydrothermal conditions, these precursor liquids rapidly crystallize into aluminosilicate phases in a wide temperature range. Synthesis using potassium based HSIL allows crystallization of aluminosilicates, including zeolites like LTL, MER, GIS, and EDI, but also dense, non-porous phases.^14,25,27^

In the current study, HSIL-based zeolite synthesis in the system 0.5SiO_2_:1KOH:4–12H_2_O:0.013Al_2_O_3_ was investigated to explore the role of water in hypo-hydrated conditions. Highly water-deficient systems were found to lead to anhydrous kalsilite polymorphs, while zeolitic phases, containing crystal water, were obtained upon slight increases of the water concentration in the syntheses. Liquid-state ^39^K, ^29^Si, and ^27^Al NMR characterization of the synthesis liquids revealed relatively small differences in (alumino)silicate speciation, but a marked change of the potassium environment and mobility. This demonstrates the profound impact of water activity on liquid phase speciation and dynamics, as well as on synthesis products. Solid products were fully characterized via chemical analysis, X-ray diffraction, scanning electron microscopy, and solid-state NMR methods. Combined, this experimental strategy enabled a detailed molecular description of the liquid and solid components of the system, revealing a direct correspondence between molecular speciation in liquid state and the synthesis products.

## 2 Results

To evaluate the influence of water on zeolite synthesis from HSIL precursors, the system SiO_2_:KOH:H_2_O:Al_2_O_3_ was selected. With only few water molecules present in the optically clear, homogeneous zeolite precursors, these hyposolvated highly alkaline systems should be considered as hydrated ionic liquids.^14,22,25^ While purely siliceous HSIL are chemically stable, addition of aluminate and thermal treatment results in crystallization of solid products owing to the limited solubility of aluminosilicate oligomers in interaction with the alkali cations. To maintain homogeneous liquid precursors in this study, the upper limit for the Al_2_O_3_/KOH ratio was chosen as Al_2_O_3_/KOH = 0.013. This way, spontaneous turbidity due to gelation or aggregate formation at room temperature can be strictly avoided. The systems had a fixed composition of 0.5SiO_2_:1KOH:*x*H_2_O:0.013Al_2_O_3_, and differed exclusively in the number of water molecules *x*, with *x* = 4, 8, and 12. These compositions and their corresponding solid products are further referred to as samples A, B, and C, respectively.

### Molecular Characterization of the Synthesis Liquids

**Figure 1:**
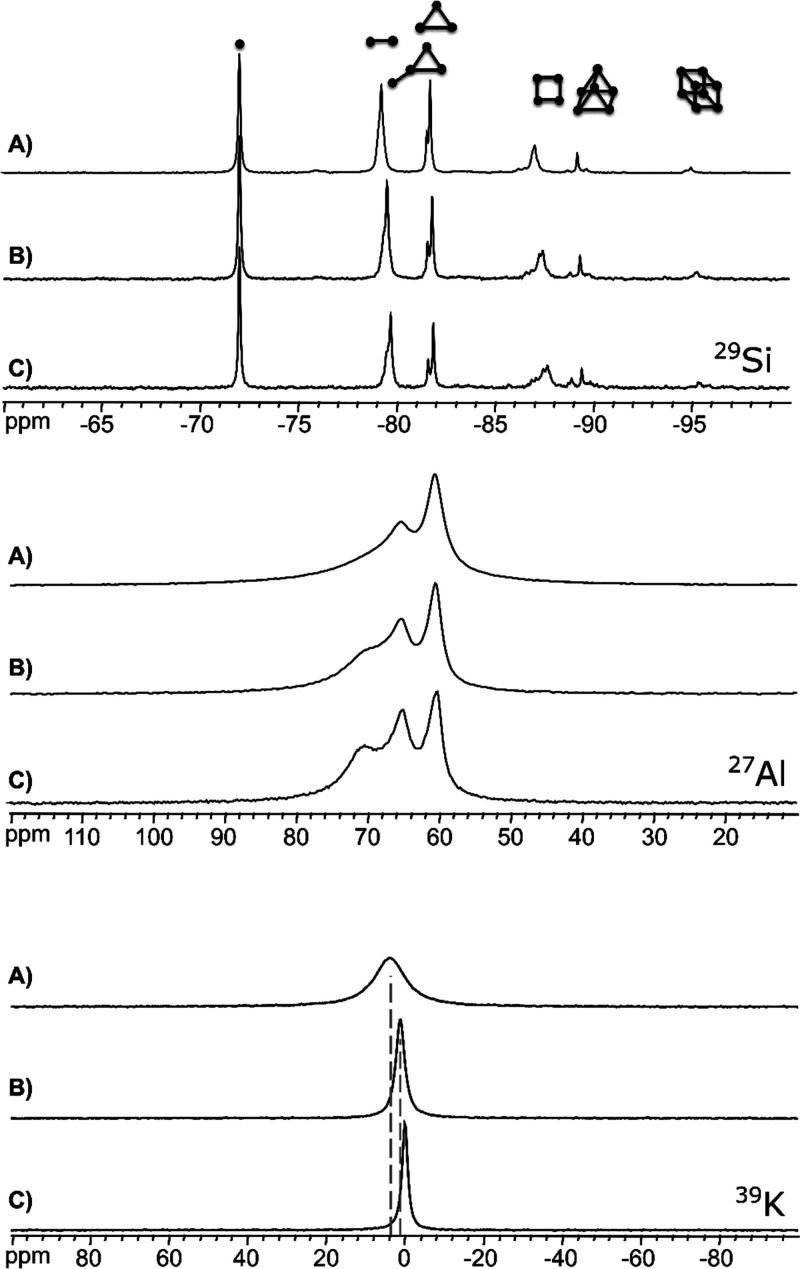
^29^Si, ^27^Al, and ^39^K NMR spectra of HSIL synthesis mixtures in the system 0.5SiO_2_:1KOH:*x*H_2_O:0.013Al_2_O_3_ with *x* equal to (A) 4, (B) 8, and (C) 12, prior to hydrothermal treatment. Silicate oligomers representative for the characteristic resonances^33,34^ in a given ppm range are illustrated in the ^29^Si NMR spectra and listed in Table S5.

To gain information about the speciation in the syntheses, the precursor liquids A, B, and C were investigated by multinuclear NMR. The studied mixtures were homogeneous liquids, demonstrated by the absence of any nanoaggregates or nanoparticles detectable by DLS or NMR (Figures 1 and S1). With further dilution and higher aluminum concentration, aluminosilicate aggregation can however be observed from the appearance of a nanosized population in the DLS analysis (see Figure S1 in the Supporting Information). Earlier, we demonstrated that even at the lower aluminate fractions, all solutions are eventually destabilized when the water content is even further increased.^14,22^

^29^Si NMR provides information on the connectivity of the (alumino)silicate oligomers in the precursor liquids. ^29^Si NMR spectra for liquids with variable water content are displayed in Figure 1. As result of the higher viscosity combined with the enhanced dynamic interconversion between the oligomers interacting and coordinating with alkali cations in the HSIL medium, the ^29^Si resonances are 1 order of magnitude broader compared to dilute aluminosilicate solutions (1 Hz).^28^ Aside from affecting the line widths, the varying water content does not affect the type of silicate species in the studied compositional range, only their relative abundance. All media exclusively contain monomeric and small oligomeric silicate species, ranging from dimers up to octamers, whose relative populations decrease with increasing nuclearity. Quantitative analysis of the spectra as a function of water content demonstrates a trend toward lower average nuclearity ⟨*n*⟩_Si_ of the oligomers with increasing water content. This is attributed to partial hydrolysis of Si–O–Si bonds at the lowered absolute silicate concentration, a process consuming water. At the same Si/OH^–^ ratio, liquids with a higher absolute silicate concentration and correspondingly low water concentration, exhibit increased condensation of silicate, a process releasing water, favored in water deprived systems. The average connectivity ⟨*n*⟩_Si_ remains in the range of 0.9 to 1.3 (Table S4). These values are estimated from the ^29^Si NMR spectra by spectral decomposition, which is exemplified in the Supporting Information (Figure S2). Characteristic oligomers were assigned to the observed resonances, illustrated in Figure 1 and listed in Table S5. Aluminosilicate resonances cannot easily be detected in the ^29^Si NMR spectra in Figure 1 due to the low concentration of aluminate in these systems. In samples containing a higher aluminum concentration, aluminosilicate resonances at −76 and −83 ppm can easily be observed. Figure S2 provides an example with composition 0.5SiO_2_:1KOH:8H_2_O:0.019Al_2_O_3_, a system still remaining in the gel- and nanoparticle-free regime.

High-aluminum zeolites or minerals easily crystallize from HSIL precursor mixtures, and the yield is limited by the availability of aluminosilicate, despite very high nominal Si-to-Al ratios in the liquid. This emphasizes the importance of aluminosilicate oligomers as the key species for the crystallization process.^14,22^ Liquid state ^27^Al-NMR was used to probe the aluminosilicate speciation in the precursor liquids (Figure 1). Despite the quadrupolar broadening,^29^ oligomeric aluminosilicates can be resolved. In general, five distinct local environments, or Q^*n*^ sites, i.e., (^−^O)_4-*n*_Al(OSi)_*n*_, exist, characterized by chemical shifts at ca. 80, 75, 70, 65, and 60 ppm for Q^0^, Q^1^, Q^2^, Q^3^, and Q^4^ respectively.^13^ In Figure 1, three resonances corresponding to Q^4^, Q^3^, and Q^2^ aluminum with respectively 4, 3, and 2 oxolated silicate neighbors^30^ can be discerned around 60, 65, and 70 ppm, respectively. Figures S3 and S4 and Table S3 in the Supporting Information show the spectral decomposition and quantitative assignments of the resonances. As can be seen in Figure 1, the spectral resolution increases upon dilution. This is related to an increase in the rate of isotropic molecular tumbling as the viscosity decreases. The average aluminum connectivity can be calculated from the weighted average of each contribution, i.e., .^31^ With increasing water content (*x*) the average Al connectivity ⟨*n*⟩_Al_ decreases slightly, with values of 3.35, 3.10, and 3.03 for *x* = 4, 8, and 12, respectively. This corresponds to the observations made for the silicate oligomers (vide supra), but the change in connectivity for Al is smaller compared to Si. The significantly higher Al connectivity ⟨*n*⟩_Al_ as compared to that of Si ⟨*n*⟩_Si_ is in line with expectations, since formation of Al–O–Si bonds is energetically favored, compared to Si–O–Si bonds.^23^

The ^39^K NMR spectra for all samples exhibit a single resonance, representing a time average of many different local environments. Both the line width and the chemical shift of this resonance strongly correlate with dilution (Figures 1 and S5). Since ^39^K is a quadrupolar nucleus (*I* = ^3^/_2_), its line width not only depends on the isotropic tumbling rate and averaging of dipolar and first order quadrupolar interactions, but also on the magnitude of the local electric field gradient (EFG). Solute concentration and ionic strength affect the sample viscosity, dynamics and diffusion behavior of potassium, while ion pairing of the cation with (alumino)silicate anions strongly influences its local environment.^32^ With increasing water content, the K^+^ signal narrows from 170 to 30 Hz and shifts downfield from 3.7 to 0.0 ppm. Increasing the availability of water increases the average coordination number of oxygen atoms from water, thus modifying the cation environment, as indicated by the change in ^39^K chemical shift toward a more shielded environment. The associated weakening of the ion-pairing strength simultaneously can be expected to enhance the kinetics of chemical exchange between potassium in different local environments, similar to what has been reported in literature for Na.^32^ Changes in the liquid-state speciation and dynamics described here for K-based HSIL, correspond well to previous observations in Na-based HSIL with analogous compositions,^22^ thus indicating the observed trends are general.

### Characterization of Solid Products

**Figure 2:**
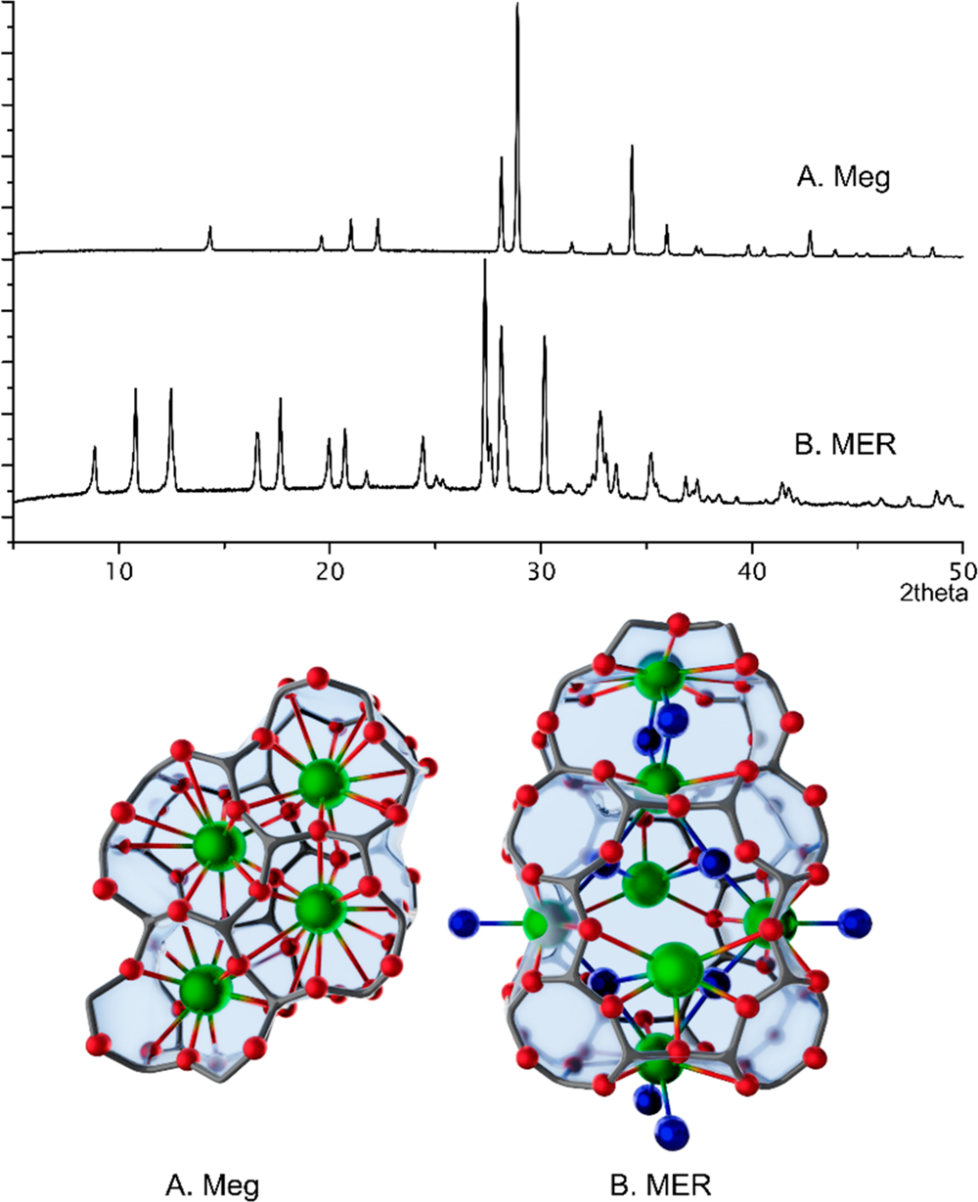
Laboratory powder XRD patterns of (A) Megakalsilite, sample A, and (B) MER, sample B, and representation of their crystal structures^35,36^ with framework oxygen, potassium and water as respectively red, green, and blue spheres. Framework T atoms are not drawn explicitly.

The examined HSIL synthesis series varies exclusively in its water content, in the range of 4 to 12 expressed as [H_2_O]/[KOH], thus changing the nominal number of water molecules available in the liquid for cation and anion coordination. At 170 °C, HSIL systems with composition 0.5SiO_2_:1KOH:*x*H_2_O:0.013Al_2_O_3_ crystallize the feldspathoid megakalsilite (Meg) at the lowest water content (*x* = 4, sample A) while MER-type zeolite is obtained at higher water content (*x* = 8, 12, samples B and C). Figure 2 shows the obtained powder XRD patterns for samples A and B. The solid phase megakalsilite and MER products were fully characterized via XRD, SEM, NMR, TGA, and ICP analysis.

#### X-ray Diffraction

The high-resolution synchrotron powder pattern of megakalsilite revealed some minor, spurious reflections, undetectable in the laboratory XRD pattern, which were assigned to a side phase or residue not removed upon rinsing of the powder after synthesis. This impurity is tentatively assigned as a KHSiO_5_ polymorph (arguments in Supporting Information, section 2). The phase assignment of megakalsilite was confirmed via Rietveld refinement (Figures S6 and S7), with starting coordinates taken from the originally published crystal structure for this mineral.^35^ Final structure coordinates and refinement parameters are reported in the Supporting Information (Tables S1 and S2). No spurious peaks were detected in MER samples, which correspond to reported diffraction patterns for zeolite W in the orthorhombic space group *Immm*.^37,38^

Megakalsilite (chemical formula KAlSiO_4_), as the name suggests, is a kalsilite polymorph. Kalsilite structures strictly show a Si/Al ratio of 1 and are constructed from parallel sheets of 6-membered rings of alternating SiO_4_ and AlO_4_ tetrahedra. The kalsilite polymorphs display different connectivity between subsequent sheets. While in kalsilite, alternating SiO_4_ and AlO_4_ tetrahedra have a consistent up–down orientation (UDUDUD), megakalsilite sheets show very specific sequences of UUUDDD and UDUDUD orientation, resulting in pocketed cages with trigonal symmetry, next to small cavities.^35^ The structure exhibits 4-rings next to 6-rings and is densely packed with potassium cations. Megakalsilite was first discovered in 2002,^35^ and only very few reports describe its synthesis. Recently, it was also obtained using a very specific aluminosilicate source, a single molecular precursor (PyH)[Al{Ph_2_Si(OSiPh_2_O)_2_}_2_] (PyH = pyridinium cation).^39^ Megakalsilite and related potassium feldspars are precursor phases for leucite production, which find applications in ceramics and porcelain-fused-to-metal systems.^40^

Zeolite W, the zeolite product obtained when increasing the water content in the same K-HSIL based synthesis^37,41^ exhibits the MER topology, which bears no structural relationship to megakalsilite. It is a small-pore zeolite with a 3-dimensional pore structure, consisting of sideways connected double crankshaft chains (dcc), forming a tetragonal net of parallel 8MR channels and characteristic *pau* cavities (Figure 2). The MER topology contains only 4- and 8-rings and is known for its cation exchange properties, porous nature and framework flexibility.^42^ Zeolite W has been identified as a selective adsorbent for Cs^+^ and Sr^2+^ radioisotopes,^43^ and as a potential candidate for CO_2_ adsorption from gas mixtures.^44^ In an earlier study,^14^ synthesis from K-based HSIL at lower temperatures produced hydrated zeolite structures for all compositions examined here. The most concentrated, Meg forming mixture (sample A) yields hydrated GIS at 90 °C, while mixtures with compositions B and C also crystallized MER.

#### Solid-State NMR

**Figure 3:**
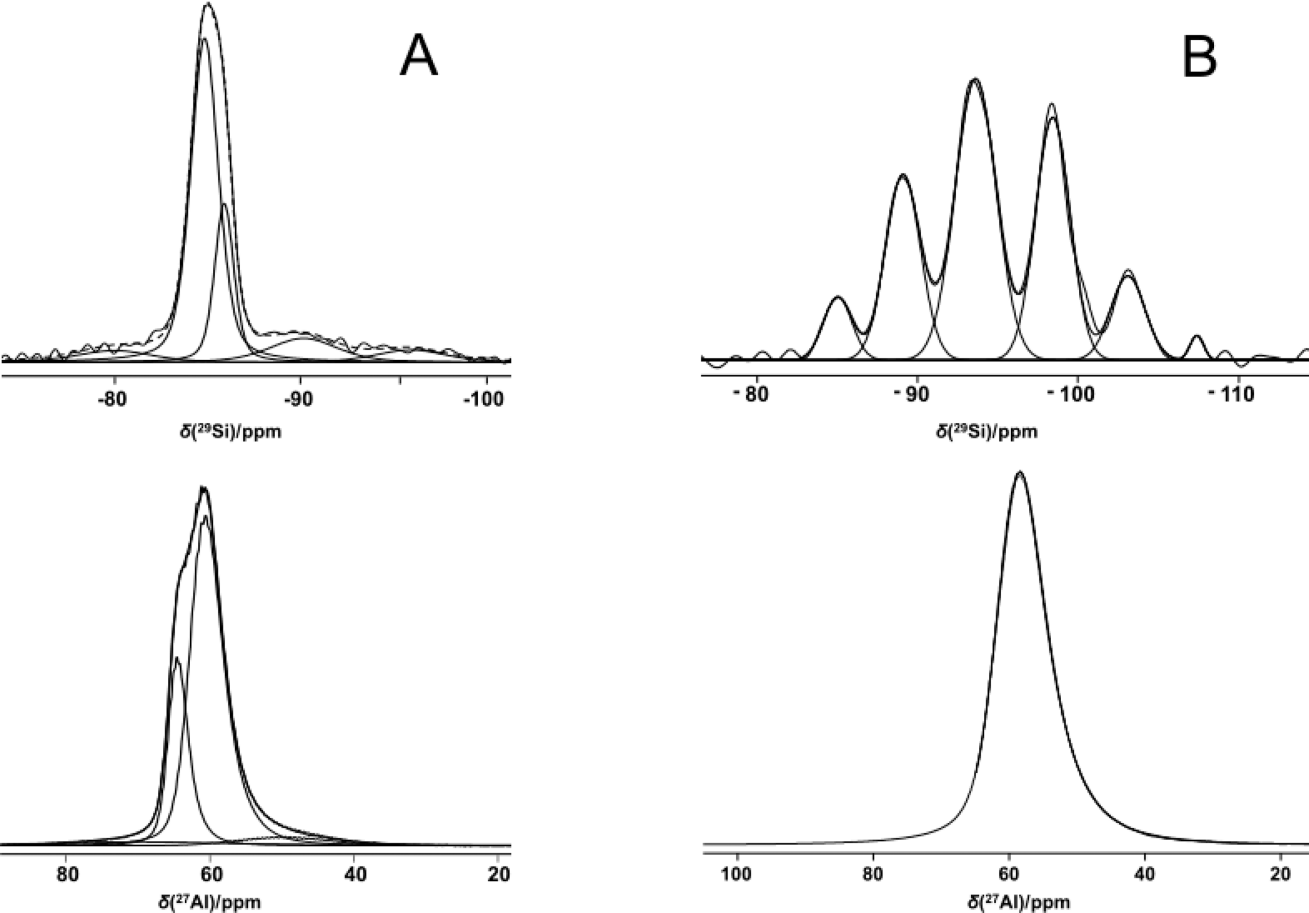
^27^Al and ^29^Si MAS NMR spectra of (A) megakalsilite, sample A, and (B) MER, sample B.

^29^Si and ^27^Al MAS NMR spectra of megakalsilite and MER samples were recorded to elucidate the local structure around the ^29^Si and ^27^Al nuclei. The ^27^Al MAS NMR spectrum of the megakalsilite sample resolves two distinct resonances in a 3:1 intensity ratio (Figure 3). Evaluating the refined crystal structure (Figure S7, Table S1), these resonances correspond to the 4 crystallographic Al sites having equal multiplicities. Three of these sites have a highly similar coordination environment resulting in superposition of their respective resonances, yielding the 3:1 ratio. Resolution of distinct crystallographic aluminum sites in aluminosilicates from the ^27^Al MAS NMR spectra is rare but can indeed be observed in case of significant Al ordering in the framework and a high degree of crystallinity.^45^

The ^29^Si MAS NMR spectrum of megakalsilite shows a main signal at −85.5 ppm, similar to what was observed by Gregorkiewitz et al. for the KAlSiO_4_–*O1* polymorph of kalsilite,^46^ and corresponds to the Q^4^(4Al) environment of the megakalsilite framework. This asymmetric resonance can be decomposed into two contributions with a 3:1 ratio, reflecting the 3:1 distribution in the T-sites already observed in the ^27^Al spectrum. The broad shoulders in the ^29^Si MAS NMR spectrum were tentatively assigned to the impurity phase, also observed in the synchrotron powder pattern of megakalsilite (supra). The range of these resonances is consistent with what has been observed for glasses with dominating potassium meta- and disilicate composition.^47,48^ In contrast with the highly ordered situation of megakalsilite, most aluminosilicates exhibit a distribution of different Al sites in the solid. This distribution in the local environment results in unresolved ^27^Al spectra,^49^ as is the case for the MER zeolites formed here. The ^29^Si MAS NMR spectrum of MER samples corresponds well to the published spectrum for zeolite W.^50^ The spectrum displays distinct resonances, assigned as overlapping signals of different Si(*n*Al) species for the two crystallographic T-sites for 0 ≤ *n* ≤ 4. The ^29^Si MAS NMR spectrum of sample C closely resembles that of sample B (supporting info Figure S9, Table S6).

#### Scanning Electron Microscopy

**Figure 4:**
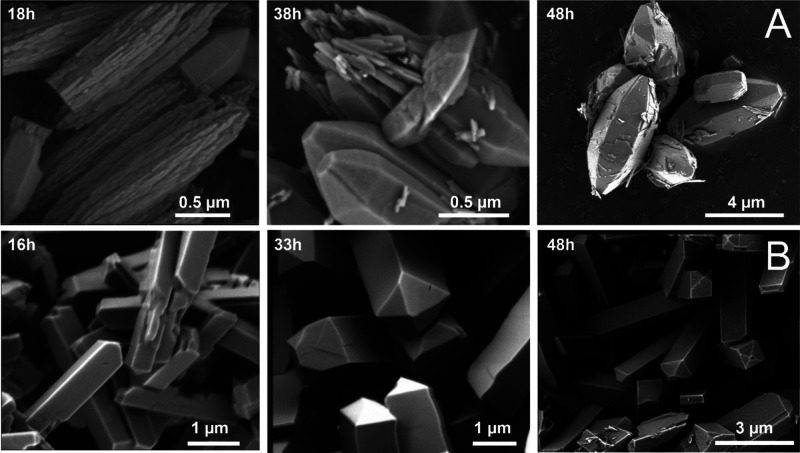
SEM images of (A) megakalsilite and (B) MER, recovered as a function of synthesis times.

SEM imaging of megakalsilite revealed micrometer-sized faceted hexagonal, bullet-shaped crystals, approximately 6 μm in length and 3 μm across after 48 h of synthesis. SEM images of samples recovered after different time intervals during synthesis reveal a peculiar growth mechanism (Figure 4). Initially, the crystals are composed of largely nonfaceted aggregates (±2.5 μm long), already displaying the macroscopic shape of the final crystals. Over time, crystal facets develop and crystal size increases, resulting in the final crystal morphology after 48 h, suggesting an Ostwald-type ripening process. The here observed morphology does not correspond to previously reported crystal habits for synthetic megakalsilite, exhibiting hexagonal or even star-shaped platelets.^51,52^ Presumably, the bullet-like morphology is a consequence of the elongated aggregates initially formed and is unique for the here described synthesis protocol. In contrast to the slow evolution of faceted Meg crystals, already after 16 h of synthesis, MER crystals have developed as cleanly faceted square rods (±3 μm in length and 0.8 μm across), a typical morphology for zeolite W.^27,38^ Again, Ostwald ripening is observed over time, as crystals increase in length and thickness, with final dimensions approximately 6 μm × 1.3 μm, with some polydispersity, while the terminal crystal facets become better defined.

#### Chemical Analysis

Framework Si/Al content of megakalsilite (*x* = 4, sample A), MER (*x* = 8, sample B), and MER (*x* = 12, sample C) were determined via ICP as 1.0, 1.9, and 2.36, respectively. For megakalsilite, this implies an ordered arrangement of alternating SiO_4_ and AlO_4_ units, respecting the Löwenstein rule. Water loss between RT and 700 °C, as measured via thermogravimetric analysis, revealed a water loss of ca. 1 and 14 wt % for megakalsilite and MER, respectively (Figure S8). Megakalsilite shows negligible weight loss before 300 °C, implying absence of any structural pore water and confirming the dense, anhydrous nature of this mineral. The small weight loss at higher temperatures can be ascribed to condensation of silanols or aluminols on the exterior crystal surface. For MER, a ca. 13 wt % loss was recorded between RT and 300 °C, attributed to loss of hydration water from the micropores.^37^ Assuming charge neutrality, compositions of solid products were thus calculated to be KAlSiO_4_ for megakalsilite and K_11_Al_11_Si_21_O_64_. 20 H_2_O for MER (sample B).

## 3 Discussion

Thermochemical studies on zeolites and minerals by Navrotsky and co-workers demonstrated porous aluminosilicate zeolites, after dehydration, to be slightly metastable (7–15 kJ/mol TO_2_) with respect to their corresponding (i.e., identical stoichiometry) anhydrous, dense minerals.^23,53^ The energy difference can readily be compensated by the hydration enthalpy of the otherwise undercoordinated cations in the zeolite pore. The energetic advantage of hydration provides porous hydrated aluminosilicates with a field of thermodynamic stability at low to moderate temperatures, compared to dense minerals and free water. However, the configurational entropy of water confined in the micropores of the zeolite framework is unfavorable as compared to that in the hydrogen bonded network in liquid water.^23,54,55^ Energetic values of hydration enthalpy and entropy for aluminosilicates are summarized and discussed in the review paper by Navrotsky et al.^23^

With increasing temperature, therefore, the relative stability of porous versus dense aluminosilicates may again be reversed as evident from the expression of the temperature dependent free energy of hydration: Δ*G*_hydr_ = Δ*H*_hydr_ – *T*Δ*S*_hydr_. In other words, the formation of porous, hydrated materials is a hydration enthalpy driven process. Upon heating in air, the unfavorable entropy of hydration results in dehydration and may lead to structural collapse and subsequent recrystallization of aluminosilicate zeolites into denser phases.^56,57^ During synthesis, similar principles apply, with additional complexity since the “net” free energy of hydration also depends on the energetic cost of removing water from the synthesis mixture.^58,59^ Unfavorable configurational entropy of confined crystal water, combined with a decrease in water activity in the synthesis mixture at high temperatures, favors aluminosilicate structures with reduced pore water content and corresponding lower porosity.^23,60^ Commonly observed framework transformations with increasing temperature and typical reductions of structural water in the crystals, are for example the LTA to SOD, or the FAU to GIS to ANA recrystallizations as a function of temperature in identical batches.^18,61^

In this study, selective discrimination between non-porous or porous aluminosilicate crystals was instead achieved at the identical synthesis temperature of 170 °C, simply by altering the water content starting from hyper-concentrated synthesis liquids. It confirms the energetic landscape governing selection of porous or non-porous crystals is more complicated when stability needs to be evaluated with respect to the synthesis medium in equilibrium with the crystal, rather than *ex situ*, i.e., removed from the mother liquor post synthesis (in air or vacuum).^62^

Recently, we demonstrated the impact of batch dilution on the Si/Al ratio of the crystallization products for different alkali cations (Na, K, and Cs) for a wide range of batch compositions at a lower synthesis temperature of 90 °C.^14^ Increasing dilution consistently resulted in crystallization of frameworks with reduced aluminum contents. Accordingly, through evaluation of the obtained framework structures and their extra-framework cation distributions in that study, we suggested a theoretical framework explaining why zeolite topologies are selective for specific Si/Al ratios.^9^ That framework, however, does not yet encompass the influence of water on the system. In this study, the decisive role of water is evidenced by the structural characterization of the solid products, as variation of the water content has a profound impact on the crystalline product formed in equilibrium with the mother liquor. Topology, framework Si/Al ratio, and water content (or porosity) of the final crystalline product were all shown to strongly depend on the water content of the precursor liquid. Increasing water levels lowers absolute concentration and activity of all liquid species, but liquid state NMR characterization (Figure 1) revealed no impactful changes in the occurrence of any specific kind of (alumino)silicate oligomers (Table S5), and only moderate changes in their relative distribution in solution (Tables S3 and S4), as the water content is varied within the here examined compositional range. Specific oligomers are therefore unlikely to be discriminating between the different observed topologies. Most striking, however, is the change of the chemical environment and mobility of the cation with increasing water content. While insights directly linking the liquid state equilibrium distribution and corresponding solid product remain scarce, the water content of the synthesis mixture was demonstrated to affect the speciation in solution, specifically the coordination environment for the charge compensating potassium cations (Figure 1). This change in liquid speciation of all solutes must in turn define the solubility and correspondingly also the Si/Al ratio of the solid product formed in equilibrium with the supernatant.^60,63,64^ In this study, this is reflected in the increase in framework Si/Al ratio when [H_2_O]/[KOH] in the precursor liquid is increased from 4 (Meg, Si/Al = 1) to 8 (MER, Si/Al = 1.9) to 12 (MER, Si/Al = 2.2). Even though not fully understood, it is plausible that one of the key reasons increased water contents have such an impactful change on the relative solubility of different products, promoting an increase in framework Si/Al, is related to the global charge density of the system. Increased dilution reduces the overall charge density of the precursor liquids, promoting crystallization of a framework with similarly reduced charge density, i.e., lower aluminum and corresponding cation contents, minimizing overall charge gradients in the entire crystallization system.

Yet, an increase in framework Si/Al ratio does not in itself explain the formation of either a porous crystal, or a dense mineral. Porous potassium aluminosilicate zeolites with Si/Al = 1 (K-LTJ,^65^ K-EDI,^66^ K-BPH),^67^ or dense minerals like leucite^68^ with Si/Al = 2 (i.e., close to the measured values of the MER products) exist as well and therefore might be expected to form at these synthesis conditions. A rational explanation can instead be derived from the differences in the liquid ^39^K-NMR spectra (Figure 1). As elaborated previously, potassium is strongly undercoordinated by water due to severe water deprivation in the precursor liquid with *x* = 4, resulting in maximal ion-pairing with aluminosilicate anions in the synthesis liquid as observed from the chemical shift and line broadening of the ^39^K-NMR spectrum. The water activity in this system is exceptionally low compared to free, liquid water, and cannot be considered a true solvent. Since all water (nominally 4 molecules per K atom) present in this synthesis liquid is either involved in silicate hydrolysis or as hydration water in the first coordination shell of the cation, incorporation as crystal water at high temperatures would be costly from an entropy perspective as it can instead be released in the solution during crystallization. This entropy gain of the liquid results in the preferential formation of an anhydrous structure, wherein each cation is fully coordinated by the framework: megakalsilite in this case. Indeed, a recent review by Gebauer documenting theoretical advancements in nucleation and crystal growth of inorganic materials from solution emphasizes the critical role of solvent configurational entropy to the free energy in all stages of crystallization, driving the solvation and association energetics of ionic solutes.^17^ The drastic sharpening of the potassium NMR signal in Figure 1 impressively documents the increased dynamics within the liquid in the presence of even few additional water molecules. Doubling the water content (*x* = 8) already shifts the potassium speciation much closer to that observed in dilute potassium silicate solutions,^32^ indicating enough water is present to solvate the cations in a dynamic hydrogen-bonded continuum. As a result, the entropic penalty for water inclusion is lower and formation of a hydrated porous crystal is promoted. Hence, hydrated MER-type zeolite crystallizes. This provides an energetic argument for the observed synthesis results.

Also from a kinetic perspective, the selective formation of dense or hydrated, porous aluminosilicates can be rationalized. Water not only affects the speciation in solution, but also the mobility and dynamics of these species. Dilution reduces the viscosity and enhances chemical exchange and interconversion processes and certainly increases the average number of water molecules in the coordination sphere of the cation. The NMR results indicate lowered viscosity and corresponding higher dynamics of the potassium complexes in the medium as evident from the sharpened ^39^K-signal. This may also explain why in the water deprived system, megakalsilite crystal facets are expressed at later times compared to the merlinoite. Faster condensation and dissolution on the nucleating and growing crystals should manifest in a faster achievement of the most stable crystal habit, as also observed by comparison of sodium with cesium zeolites obtained from HSIL.^2^ In hydrated zeolites, water is necessary to separate adjacent cations, effectively shielding their positive charges, allowing them to occupy positions which only are partially coordinated by the framework. Water molecules already associated with the cations in the synthesis medium may play a similar role, allowing a cation–water distribution around which condensation of T atom facilitates formation of a porous framework. In contrast, when no water is available to separate neighboring cations, they need to be separated by framework species on all sides, enforcing a dense anhydrous framework.

## 4 Conclusion

Hyposolvated hydrated silicate ionic liquids (HSIL) were exploited to investigate the role of water in zeolite synthesis. Synthesis liquids with a fixed chemical composition were studied, varying exclusively the concentration of water in systems with 0.5SiO_2_:1KOH:*x*H_2_O:0.013Al_2_O_3_, *x* ranging from 4 to 12. The liquid-state speciation of silicate, aluminate and potassium was examined via ^29^Si, ^27^Al, and ^39^K NMR, revealing the exclusive presence of small oligomeric silicates and aluminosilicates. As a function of water content, increased dynamics of the ion-paired clusters was observed with increasing hydration.

Hydrothermal treatment at 170 °C for 48 h produced zeolite MER for H_2_O/KOH = 8–12. At the same temperature, a lower amount of water in the synthesis (H_2_O/KOH = 4) yielded megakalsilite, a non-porous anhydrous potassium aluminosilicate KAlSiO_4_. The products were fully characterized by powder XRD, solid state NMR, TGA, SEM, and ICP analysis. Asides expression of different topologies also the Si/Al ratio was affected by the water content of the precursor liquid: 1.9–2.4 in the case of MER and 1.0 for megakalsilite, although the Si/Al and SiO_2_/KOH ratios are strictly identical in the initial synthesis liquids. This difference can be related to the solubility of the respective materials in the mother liquor, which is a function of the composition and charge density of the batch.^62−64,69^

Phase selectivity in these systems is immediately impacted by the availability of water, directing the synthesis toward either porous or dense phases. The observed results were explained in terms of both the thermodynamic implications of cation hydration and the molecular mobility and ion-association of the cation in the precursor liquid. In a water-deficient system, the cation hydration entropy upon inclusion in a microporous framework is excessively expensive. This thermodynamically favors dense anhydrous solids over hydrated porous materials. On the other hand, when enough water is present to sufficiently solvate the cations in synthesis medium and solid, the net entropy gained from releasing a few extra water molecules into solution becomes smaller. Therefore, porous frameworks crystallize, energetically driven by the favorable hydration enthalpy of the coordination water in the pore system.

## 5 Experimental Section

### Preparation of Silicate Ionic Liquid (HSIL) Precursor

In a typical procedure, hydrated silicate ionic liquids, i.e., hyposolvated liquid alkali-silicate,^25,27^ are prepared via hydrolysis of TEOS (Si(OEt)_4_, ACROS, 98+%) under vigorous magnetic stirring by KOH (Fisher Scientific 85+%) in the global system 1 TEOS: 1 KOH: 20 H_2_O (Milli-Q ultrapure water). After complete TEOS hydrolysis, spontaneous liquid–liquid phase separation occurs. This yields chemical compositions gravimetrically and NMR-wise determined to consist of an upper layer with 4EtOH:12H_2_O composition and a dense lower layer with 1SiO_2_:1KOH:6H_2_O composition. Using a separating funnel, the silicate containing fraction is easily collected and readily available for use as a silicon source in zeolite synthesis.

### Hydrothermal Synthesis

The experimental procedure for preparation of the precursor mixtures is analogous to that reported in a previous article.^27^ A concentrated alkaline aluminate solution was prepared by fully dissolving amorphous Al(OH)_3_ (Aldrich, 50–57.5 wt % as Al_2_O_3_) in a KOH solution, with final composition 1KOH–3H_2_O–0.05Al_2_O_3_. The native HSIL was combined with appropriate amounts of KOH and H_2_O, and the aluminate solution was added dropwise while vigorously stirring. The resulting precursor mixtures were aged in a closed vessel under agitation for at least 1 h, yielding homogeneous, transparent liquids with composition 0.5SiO_2_:1KOH:*x*H_2_O:0.013Al_2_O_3_, *x* = 4, 8, or 12. These synthesis mixtures were subsequently transferred to PTFE liners and autoclaved in a tumbling oven at 170 °C for reaction times ranging from 16 to 48 h. The crystals were collected and rinsed via repeated centrifugation–redispersion and dried in air at 60 °C prior to characterization.

### Characterization of HSIL Precursor Liquids

#### Liquid State NMR

Liquid state characterization was carried out approximately 24 h after addition of the aluminate to the native HSIL and aging at room temperature. HSIL precursors liquids were analyzed by ^27^Al, ^29^Si and ^39^K NMR at room temperature (26 °C). ^27^Al and ^29^Si NMR experiments were carried out on a Bruker Avance 500 spectrometer, operating at 130.326 MHz for ^27^Al and 99.353 MHz for ^29^Si. Direct excitation ^29^Si spectra were recorded using a π/4 pulse of 3.6 μs, a recycle delay of 5 s, and an acquisition time of 1.6 s and accumulating 4096 transients. By using a Si-background-free probe in combination with 10 mm quartz tubes, the short repetition delays enabled to saturate the background signal resulting from quartz. Direct excitation ^27^Al NMR spectra were obtained by accumulating 1024 transients using a π/12 excitation pulse of 1.9 μs, an acquisition time of 0.4 s and a recycle delay of 0.1 s. ^39^K NMR spectra were recorded on a Bruker Avance 400 spectrometer operating at 18.672 MHz, with a pulse of 9.1 μs (π/8), a recycle delay of 0.1 s, an acquisition time of 0.5 s, and an accumulation of 1024 scans. Spectra were referenced to tetramethylsilane (TMS) for ^29^Si, 1 M Al(NO_3_)_3_ in water for ^27^Al, and 2 M KCl in water for ^39^K.

#### Dynamic Light Scattering

DLS analysis of the HSIL precursors was performed on a ALV/CGS-3 instrument (ALV, Langen, Germany) using 60 s measurements at scattering angles from 30° to 150° at a wavelength of 632.8 nm.

### Characterization of Solid Products

#### X-ray Diffraction

Laboratory PXRD patterns (Cu Kα_1_ radiation) were recorded at room temperature on a STOE STADI MP diffractometer with focusing Ge(111) monochromator in Debye–Scherrer geometry (0.5 mm capillary), with a linear position sensitive detector (internal resolution 0.01°). A synchrotron powder X-ray diffraction (PXRD) pattern of megakalsilite was collected at the Crystal beamline at Soleil (λ = 0.62236 Å) using a dried powder sample (0.7 mm capillary).

#### Solid State NMR

^27^Al and ^29^Si Solid-state NMR experiments were performed on Bruker 300 MHz Avance III spectrometer operating at magnetic field strength of 7.05 T equipped with a 4 mm ^1^H/X double resonance probehead. Hydrated samples were packed in 4 mm zirconia rotors and spun at 15 kHz. Direct excitation ^27^Al (78.2 MHz) MAS NMR measurements were carried out using a central-transition (CT) selective excitation pulse (π/12) of 75 kHz RF field strength and SPINAL64^70^^1^H decoupling at 56 kHz RF field strength, accumulating 1024 scans with a recycle delay of 1 s. Direct excitation ^29^Si NMR spectrum was recorded with a π/2 pulse of 66 kHz RF field-strength and 40 kHz ^1^H SPINAL-64 decupling. 600 transients with repetition delay of 1600 s and 320 transients with repetition delay of 1000 s were acquired for the megakalsilite and MER respectively. ^27^Al NMR spectra were referenced to 0.1 M Al(NO_3_)_3_ in D_2_O. ^29^Si NMR spectra were referenced to secondary reference, Q8M8, which was further referenced to the primary reference, TMS. All the spectra were decomposed with DMFIT.^71^

#### Scanning Electron Microscopy

SEM images were recorded on a Nova NanoSEM450 (FEI, Hillsboro, OR).

#### Thermogravimetric Analysis

TGA was performed on a TGA Q500 (TA Instruments) under N_2_ flow (10 mL/min) with a heating rate of 2 °C/min between 25 and 700 °C.

#### Elemental Analysis

Si and Al contents were determined from dissolved samples on an axial simultaneous ICP-OES instrument (Varian 720-ES) with cooled cone interface and oxygen-free optics. Samples for ICP were prepared by digesting 50 mg of zeolite powder with 250 mg of LiBO_2_ in a muffle furnace at 1000 °C prior to diluting with 0.42 N HNO_3_ solution.

## Supporting Information Available

Liquid- and solid-state characterization as referenced in the main text, DLS data, NMR spectra, Rietveld refinement figures, structural data of megakalsilite, and thermogravimetric analysis (PDF)

## References

[ref1] Barrer R. M. Zeolites and Their Synthesis. Zeolites 1981, 1, 130–140. 10.1016/S0144-2449(81)80001-2.

[ref2] Pellens N.; Doppelhammer N.; Asselman K.; Thijs B.; Jakoby B.; Reichel E. K.; Taulelle F.; Martens J.; Breynaert E.; Kirschhock C. E. A. A Zeolite Crystallisation Model Confirmed by In-Situ Observation. Faraday Discuss. 2022, 235, 162–182. 10.1039/D1FD00093D. 35660805

[ref3] Price S.; Rimez B.; Sun W.; Peters B.; Christenson H.; Hughes C.; Sun C. C.; Veesler S.; Pan H.; Brandel C.; Biscans B.; Meekes H.; Rosbottom I.; Roth W. J.; Seton L.; Taulelle F.; Black S.; Threlfall T.; Vekilov P.; Poornachary S.; Diemand J.; Toroz D.; Salvalaglio M.; Tipduangta P.; Sefcik J.; Booth S.; Rasmuson A.; Janbon S.; ter Horst J.; Simone E.; Hammond R.; Bertran C. A.; Vetter T.; Sear R.; de Yoreo J.; Harris K.; Ristic R.; Kavanagh A.; Roberts K.; Breynaert E.; Myerson A.; Coquerel G.; Wu D.; Cölfen H.; Cuppen H.; Smets M.; Wu D. T. Nucleation in Complex Multi-Component and Multi-Phase Systems: General Discussion. Faraday Discuss. 2015, 179, 503–542. 10.1039/C5FD90039E. 26081969

[ref4] Gebauer D.; Kellermeier M.; Gale J. D.; Bergström L.; Cölfen H. Pre-Nucleation Clusters as Solute Precursors in Crystallisation. Chem. Soc. Rev. 2014, 43, 2348–2371. 10.1039/C3CS60451A. 24457316

[ref5] Cundy C. S.; Cox P. A. The Hydrothermal Synthesis of Zeolites: Precursors, Intermediates and Reaction Mechanism. Microporous Mesoporous Mater. 2005, 82 (1–2), 1–78. 10.1016/j.micromeso.2005.02.016.

[ref6] Weller M. T. Where Zeolites and Oxides Merge: Semi-Condensed Tetrahedral Frameworks. J. Chem. Soc., Dalton Trans. 2000, (23), 4227–4240. 10.1039/b003800h.

[ref7] Férey G.; Haouas M.; Loiseau T.; Taulelle F. Nanoporous Solids: How Do They Form? An in Situ Approach. Chem. Mater. 2014, 26 (1), 299–309. 10.1021/cm4019875.

[ref8] van Tendeloo L.; Gobechiya E.; Breynaert E.; Martens J. A.; Kirschhock C. E. A. Alkaline Cations Directing the Transformation of FAU Zeolites into Five Different Framework Types. Chem. Commun. 2013, 49 (100), 11737–11739. 10.1039/c3cc47292b. 24202181

[ref9] Asselman K.; Vandenabeele D.; Pellens N.; Doppelhammer N.; Kirschhock C. E. A.; Breynaert E. Structural Aspects Affecting Phase Selection in Inorganic Zeolite Synthesis. Chem. Mater. 2022, 34 (24), 11081–11092. 10.1021/acs.chemmater.2c03204. 36590702 PMC9798827

[ref10] Guo P.; Shin J.; Greenaway A. G.; Min J. G.; Su J.; Choi H. J.; Liu L.; Cox P. A.; Hong S. B.; Wright P. A.; Zou X. A Zeolite Family with Expanding Structural Complexity and Embedded Isoreticular Structures. Nature 2015, 524 (7563), 74–78. 10.1038/nature14575. 26176918

[ref11] Aerts A.; Follens L. R. A.; Haouas M.; Caremans T. P.; Delsuc M. A.; Loppinet B.; Vermant J.; Goderis B.; Taulelle F.; Martens J. A.; Kirschhock C. E. A. Combined NMR, SAXS, and DLS Study of Concentrated Clear Solutions Used in Silicalite-1 Zeolite Synthesis. Chem. Mater. 2007, 19 (14), 3448–3454. 10.1021/cm070693j.

[ref12] Rivas-Cardona A.; Chovanetz M.; Shantz D. F. A Systematic Investigation of Silicalite-1 Precursor Mixtures with Varying Degrees of Dilution. Microporous Mesoporous Mater. 2012, 155, 56–64. 10.1016/j.micromeso.2011.12.048.

[ref13] Kinrade S. D.; Swaddle T. W. Direct Detection of Aluminosilicate Species in Aqueous Solution by Silicon-29 and Aluminum-27 NMR Spectroscopy. Inorg. Chem. 1989, 28 (10), 1952–1954. 10.1021/ic00309a036.

[ref14] Asselman K.; Pellens N.; Thijs B.; Doppelhammer N.; Haouas M.; Taulelle F.; Martens J. A.; Breynaert E.; Kirschhock C. E. A. Ion-Pairs in Aluminosilicate-Alkali Synthesis Liquids Determine Aluminium Content and Topology of Crystallizing Zeolites. Chem. Mater. 2022, 34 (16), 7150–7158. 10.1021/acs.chemmater.2c00773. 36032556 PMC9404546

[ref15] Prasad D.; Mitra N. Catalytic Behavior of Hydrogen Bonded Water in Oligomerization of Silicates. Inorg. Chem. 2023, 62 (4), 1423–1436. 10.1021/acs.inorgchem.2c03509. 36657385

[ref16] Mousavi S. F.; Jafari M.; Kazemimoghadam M.; Mohammadi T. Template Free Crystallization of Zeolite Rho via Hydrothermal Synthesis: Effects of Synthesis Time, Synthesis Temperature, Water Content and Alkalinity. Ceram. Int. 2013, 39 (6), 7149–7158. 10.1016/j.ceramint.2013.02.058.

[ref17] Gebauer D.; Gale J. D.; Cölfen H. Crystal Nucleation and Growth of Inorganic Ionic Materials from Aqueous Solution: Selected Recent Developments, and Implications. Small 2022, 18, 2107735–2107748. 10.1002/smll.202107735. 35678091

[ref18] Maldonado M.; Oleksiak M. D.; Chinta S.; Rimer J. D. Controlling Crystal Polymorphism in Organic-Free Synthesis of Na- Zeolites. J. Am. Chem. Soc. 2013, 135 (7), 2641–2652. 10.1021/ja3105939. 23265176

[ref19] Valtchev V. P.; Tosheva L.; Bozhilov K. N. Synthesis of Zeolite Nanocrystals at Room Temperature. Langmuir 2005, 21 (23), 10724–10729. 10.1021/la050323e. 16262343

[ref20] Dai F. Y.; Suzuki M.; Takahashi H.; Saito Y. Mechanism of Zeolite Crystallization without Using Template Reagents of Organic Bases. Stud. Surf. Sci. Catal. 1986, 28 (C), 223–230. 10.1016/S0167-2991(09)60877-6.

[ref21] Bushuev Y. G.; Sastre G.; de Julián-Ortiz J. V. The Structural Directing Role of Water and Hydroxyl Groups in the Synthesis of Beta Zeolite Polymorphs. J. Phys. Chem. C 2010, 114 (1), 345–356. 10.1021/jp907694g.

[ref22] Pellens N.; Doppelhammer N.; Radhakrishnan S.; Asselman K.; Chandran C. V.; Vandenabeele D.; Jakoby B.; Martens J. A.; Taulelle F.; Reichel E. K.; Breynaert E.; Kirschhock C. E. A. Nucleation of Porous Crystals from Ion-Paired Pre-Nucleation Clusters. Chem. Mater. 2022, 34 (16), 7139–7149. 10.1021/acs.chemmater.2c00418. 36032557 PMC9404542

[ref23] Navrotsky A.; Trofymluk O.; Levchenko A. A. Thermochemistry of Microporous and Mesoporous Materials. Chem. Rev. 2009, 109 (9), 3885–3902. 10.1021/cr800495t. 19637927

[ref24] Zhou W.; Sun P.; Navrotsky A.; Kim S. H.; Hong S. B. Formation and Dehydration Enthalpies of Gallosilicate Materials with Different Framework Topologies and Ga Contents. Microporous Mesoporous Mater. 2009, 121 (1–3), 200–207. 10.1016/j.micromeso.2009.02.001.

[ref25] van Tendeloo L.; Haouas M.; Martens J. A.; Kirschhock C. E. A.; Breynaert E.; Taulelle F. Zeolite Synthesis in Hydrated Silicate Ionic Liquids. Faraday Discuss. 2015, 179, 437–449. 10.1039/C4FD00234B. 25886652

[ref26] Doppelhammer N.; Pellens N.; Martens J.; Kirschhock C. E. A.; Jakoby B.; Reichel E. K. Moving Electrode Impedance Spectroscopy for Accurate Conductivity Measurements of Corrosive Ionic Media. ACS Sens. 2020, 5 (11), 3392–3397. 10.1021/acssensors.0c01465. 33107724 PMC7706010

[ref27] Haouas M.; Lakiss L.; Martineau C.; el Fallah J.; Valtchev V.; Taulelle F. Silicate Ionic Liquid Synthesis of Zeolite Merlinoite: Crystal Size Control from Crystalline Nanoaggregates to Micron-Sized Single-Crystals. Microporous Mesoporous Mater. 2014, 198, 35–44. 10.1016/j.micromeso.2014.07.011.

[ref28] Kinrade S. D.; Swaddle T. W. Silicon-29 NMR Studies of Aqueous Silicate Solutions. 2. Transverse 29Si Relaxation and the Kinetics and Mechanism of Silicate Polymerization. Inorg. Chem. 1988, 27 (23), 4259–4264. 10.1021/ic00296a035.

[ref29] Haouas M.; Taulelle F.; Martineau C. Recent Advances in Application of 27Al NMR Spectroscopy to Materials Science. Prog. Nucl. Magn. Reson. Spectrosc. 2016, 94–95, 11–36. 10.1016/j.pnmrs.2016.01.003. 27247283

[ref30] Mortlock R. F.; Bell A. T.; Radke C. J. NMR Investigations of Tetrapropylammonium Aluminosilicate and Borosilicate Solutions. J. Phys. Chem. 1991, 95 (1), 372–378. 10.1021/j100154a067.

[ref31] Eilertsen E. A.; Haouas M.; Pinar A. B.; Hould N. D.; Lobo R. F.; Lillerud K. P.; Taulelle F. NMR and SAXS Analysis of Connectivity of Aluminum and Silicon Atoms in the Clear Sol Precursor of SSZ-13 Zeolite. Chem. Mater. 2012, 24 (3), 571–578. 10.1021/cm2032515.

[ref32] McCormick A. v; Bell A. T.; Radke C. J. Evidence from Alkali-NMR Spectroscopy for Ion Pairing in Alkaline Silicate Solutions. J. Phys. Chem. 1989, 93 (5), 1733–1737. 10.1021/j100342a013.

[ref33] Goudarzi N.; Chamjangali M. A.; Amin A. H.; Goodarzi M. Effects of Surfactant and Polyelectrolyte on Distribution of Silicate Species in Alkaline Aqueous Tetraoctylammonium Silicate Solutions Using 29Si NMR Spectroscopy. Appl. Magn. Reson. 2013, 44 (9), 1095–1103. 10.1007/s00723-013-0467-5.

[ref34] Haouas M.; Taulelle F. Revisiting the Identification of Structural Units in Aqueous Silicate Solutions by Two-Dimensional Silicon-29 INADEQUATE. J. Phys. Chem. B 2006, 110 (7), 3007–3014. 10.1021/jp0557823. 16494302

[ref35] Khomyakov A. P.; Nechelyustov G. N.; Sokolova E.; Bonaccorsi E.; Merlino S.; Pasero M. Megakalsilite, a New Polymorph of KAISiO4 from the Khibina Alkaline Massif, Kola Peninsula, Russia: Mineral Description and Crystal Structure. Can. Mineral. 2002, 40 (3), 961–970. 10.2113/gscanmin.40.3.961.

[ref36] Pakhomova A. S.; Armbruster T.; Krivovichev S. v.; Yakovenchuk V. N. Dehydration of the Zeolite Merlinoite from the Khibiny Massif, Russia: An in Situ Temperature-Dependent Single-Crystal X-Ray Study. Eur. J. of Mineral. 2014, 26 (3), 371–380. 10.1127/0935-1221/2014/0026-2380.

[ref37] Skofteland B. M.; Ellestad O. H.; Lillerud K. P. Potassium Merlinoite: Crystallization, Structural and Thermal Properties. Microporous Mesoporous Mater. 2001, 43 (1), 61–71. 10.1016/S1387-1811(00)00347-4.

[ref38] Houlleberghs M.; Breynaert E.; Asselman K.; Vaneeckhaute E.; Radhakrishnan S.; Anderson M. W.; Taulelle F.; Haouas M.; Martens J. A.; Kirschhock C. E. A. Evolution of the Crystal Growth Mechanism of Zeolite W (MER) with Temperature. Microporous Mesoporous Mater. 2019, 274, 379–384. 10.1016/j.micromeso.2018.09.012.

[ref39] Imaizumi A.; Nakada A.; Matsumoto T.; Chang H. C. Facile and Selective Synthesis of Zeolites L and W from a Single-Source Heptanuclear Aluminosilicate Precursor. CrystEngComm 2020, 22 (35), 5862–5870. 10.1039/D0CE00546K.

[ref40] Zhang Y.; Lv M.; Chen D.; Wu J. Leucite Crystallization Kinetics with Kalsilite as a Transition Phase. Mater. Lett. 2007, 61 (14–15), 2978–2981. 10.1016/j.matlet.2006.10.057.

[ref41] Chawla A.; Mallette A. J.; Jain R.; Le N.; Robles Hernández F. C.; Rimer J. D. Crystallization of Potassium-Zeolites in Organic-Free Media. Microporous Mesoporous Mater. 2022, 341, 112026 10.1016/j.micromeso.2022.112026.

[ref42] Choi H. J.; Jo D.; Min J. G.; Hong S. B. The Origin of Selective Adsorption of CO2 on Merlinoite Zeolites. Angew. Chem. - Int. Ed. 2021, 60 (8), 4307–4314. 10.1002/anie.202012953. 33089637

[ref43] Kakutani Y.; Weerachawanasak P.; Hirata Y.; Sano M.; Suzuki T.; Miyake T. K-Merlinoite or, ab. Highly Effective K-Merlinoite Adsorbent for Removal of Cs + and Sr 2+ in Aqueous Solution. RSC Adv. 2017, 7 (49), 30919–30928. 10.1039/C7RA03867D.

[ref44] Georgieva V. M.; Bruce E. L.; Verbraeken M. C.; Scott A. R.; Casteel W. J.; Brandani S.; Wright P. A. Triggered Gate Opening and Breathing Effects during Selective CO2 Adsorption by Merlinoite Zeolite. J. Am. Chem. Soc. 2019, 141 (32), 12744–12759. 10.1021/jacs.9b05539. 31373800

[ref45] Asselman K.; Radhakrishnan S.; Pellens N.; Chandran C. V.; Houlleberghs M.; Xu Y.; Martens J. A.; Sree S. P.; Kirschhock C. E. A.; Breynaert E. HSIL-Based Synthesis of Ultracrystalline K,Na-JBW, a Zeolite Exhibiting Exceptional Framework Ordering and Flexibility. Chem. Mater. 2022, 34 (16), 7159–7166. 10.1021/acs.chemmater.2c01059. 36032550 PMC9404536

[ref46] Gregorkiewitz M.; Li Y.; White T. J.; Withers R. L.; Sobrados I. The Structure of “Orthorhombic” KAlSiO4-O1: Evidence for Al-Si Order from MAS NMR Data Combined with Rietveld Refinement and Electron Microscopy. Can. Mineral. 2008, 46 (6), 1511–1526. 10.3749/canmin.46.6.1511.

[ref47] Davis M. C.; Kaseman D. C.; Parvani S. M.; Sanders K. J.; Grandinetti P. J.; Massiot D.; Florian P. Q(n) Species Distribution in K2Oa2SiO2 Glass by 29Si Magic Angle Flipping NMR. J. Phys. Chem. A 2010, 114 (17), 5503–5508. 10.1021/jp100530m. 20377177

[ref48] Stebbins J. F. Anionic Speciation in Sodium and Potassium Silicate Glasses near the Metasilicate ([Na,K]2SiO3) Composition: 29Si, 17O, and 23Na MAS NMR. J. of Non-Cryst. Solids: X 2020, 6, 100049 10.1016/j.nocx.2020.100049.

[ref49] Czjzek G.; Fink J.; Götz F.; Schmidt H.; Coey J. M. D.; Rebouillat J. P.; Liénard A. Atomic Coordination and the Distribution of Electric Field Gradients in Amorphous Solids. Phys. Rev. B 1981, 23 (6), 2513–2530. 10.1103/PhysRevB.23.2513.

[ref50] Kennedy G. J.; Afeworki M.; Hong S. B. Probing the Non-Random Aluminum Distribution in Zeolite Merlinoite with Ultra-High-Field (18.8 T) 27 Al and 29Si MAS NMR. Microporous Mesoporous Mater. 2002, 52 (1), 55–59. 10.1016/S1387-1811(02)00278-0.

[ref51] Yuan J.; Ma H.; Luo Z.; Ma X.; Guo Q. Synthesis of Kalsio4 by Hydrothermal Processing on Biotite Syenite and Dissolution Reaction Kinetics. Minerals 2021, 11 (1), 36 10.3390/min11010036.

[ref52] Clark McGuire M.; Bull I.; Mark Johnson G. Synthetic Megakalsilite via Hydrothermal Preparation, US 2014/0256866A1, 2014.

[ref53] Navrotsky A.; Tian Z. R. Systematics in the Enthalpies of Formation of Anhydrous Aluminosilicate Zeolites, Glasses, and Dense Phases. Chem. Eur. J. 2001, 7 (4), 769–774. 10.1002/1521-3765(20010216)7:4<769::AID-CHEM769>3.0.CO;2-J. 11288866

[ref54] Mizota T.; Petrova N. L.; Nakayama N. Entropy of Zeolitic Water. Journal of Thermal Analysis and Calorimetry 2001 64:1 2001, 64 (1), 211–217. 10.1023/A:1011549432295.

[ref55] Newton R. C.; Wood B. J. Thermodynamics of Water in Cordierite and Some Petrologic Consequences of Cordierite as a Hydrous Phase. Contributions to Mineralogy and Petrology 1979 68:4 1979, 68 (4), 391–405. 10.1007/BF01164524.

[ref56] Mimura H.; Akiba K.; Ishiyama S.; Eto M. Physical and Chemical Properties of Solid Forms Fixing Heat-Generating Nuclides. J. Nucl. Sci. Technol. 1996, 33 (6), 511–518. 10.1080/18811248.1996.9731944.

[ref57] Simancas R.; Chokkalingam A.; Elangovan S. P.; Liu Z.; Sano T.; Iyoki K.; Wakihara T.; Okubo T. Recent Progress in the Improvement of Hydrothermal Stability of Zeolites. Chem. Sci. 2021, 12 (22), 7677–7695. 10.1039/D1SC01179K. 34168820 PMC8188473

[ref58] Hellström M.; Behler J. Structure of Aqueous NaOH Solutions: Insights from Neural-Network-Based Molecular Dynamics Simulations. Phys. Chem. Chem. Phys. 2017, 19 (1), 82–96. 10.1039/C6CP06547C. 27805193

[ref59] Asselman K.; Pellens N.; Radhakrishnan S.; Chandran C. V.; Martens J.; Taulelle F.; Verstraelen T.; Hellström M.; Breynaert E.; Kirschhock C. E. A. Super-Ions of Sodium Cations with Hydrated Hydroxide Anions: Inorganic Structure-Directing Agents in Zeolite Synthesis. Mater. Horiz 2021, 8, 2576–2583. 10.1039/D1MH00733E. 34870303

[ref60] Bouchiba N.; Guzman Castillo M. L. A.; Bengueddach A.; Fajula F.; di Renzo F. Zeolite Metastability as a Function of the Composition of the Surrounding Solution: The Case of Faujasite and Zeolite Omega. Microporous Mesoporous Mater. 2011, 144 (1–3), 195–199. 10.1016/j.micromeso.2011.04.015.

[ref61] Gualtieri A.; Norby P.; Artioli G.; Hanson J. Kinetic Study of Hydroxysodalite Formation from Natural Kaolinites by Time-Resolved Synchrotron Powder Diffraction. Microporous Mater. 1997, 9 (3–4), 189–201. 10.1016/S0927-6513(96)00111-3.

[ref62] Asselman K.; Vandenabeele D.; Pellens N.; Doppelhammer N.; Kirschhock C. E. A.; Breynaert E. Structural Aspects Affecting Phase Selection in Inorganic Zeolite Synthesis. Chem. Mater. 2022, 34, 11081 10.1021/acs.chemmater.2c03204. 36590702 PMC9798827

[ref63] Šefčík J.; McCormick A. v. What Is the Solubility of Zeolite A?. Microporous Mater. 1997, 10 (4–6), 173–179. 10.1016/S0927-6513(97)00007-2.

[ref64] Šefčík J.; McCormick A. v. Prediction of Crystallization Diagrams for Synthesis of Zeolites. Chem. Eng. Sci. 1999, 54 (15–16), 3513–3519. 10.1016/S0009-2509(98)00522-3.

[ref65] Broach R. W.; Kirchner R. M. Structures of the K+ and NH4+ Forms of Linde J. Microporous Mesoporous Mater. 2011, 143 (2–3), 398–400. 10.1016/j.micromeso.2011.03.025.

[ref66] Tambuyzer E.; Bosmans H. J. The Crystal Structure of Synthetic Zeolite K-F. Acta Cryst. Section B 1976, 32B (6), 1714–1719. 10.1107/S0567740876006286.

[ref67] Andries K. J.; Bosmans H. J.; Grobet P. J. The Crystal Structure of Zeolite Linde Q: A Proposal Based on Powder X-Ray Diffraction and 27Al and 29Si MAS n.m.r. Spectroscopy. Zeolites 1991, 11 (2), 124–131. 10.1016/0144-2449(91)80405-O.

[ref68] Mazzi F. The Crystal Structure of Tetragonal Leucite. Am. Mineral. 1976, 61, 108–115.

[ref69] Guzman Castillo M. L.; di Renzo F.; Fajula F.; Bousquet J. Crystallization Kinetics of Zeolite Omega, the Synthetic Analog of Mazzite. Microporous Mesoporous Mater. 2006, 90 (1–3), 221–228. 10.1016/j.micromeso.2005.10.022.

[ref70] Fung B. M.; Khitrin A. K.; Ermolaev K. An Improved Broadband Decoupling Sequence for Liquid Crystals and Solids. J. Magn. Reson. 2000, 142 (1), 97–101. 10.1006/jmre.1999.1896. 10617439

[ref71] Massiot D.; Fayon F.; Capron M.; King I.; le Calvé S.; Alonso B.; Durand J. O.; Bujoli B.; Gan Z.; Hoatson G. Modelling One- and Two-Dimensional Solid-State NMR Spectra. Magn. Reson. Chem. 2002, 40 (1), 70–76. 10.1002/mrc.984.

