# Supplementary material for: Does Water Enable Porosity in Aluminosilicate Zeolites? Porous Frameworks versus Dense Minerals
Source: Cryst Growth Des. 2023 Mar 24;23(5):3338–48. doi: 10.1021/acs.cgd.2c01476 (PMC10161221; doi:10.1021/acs.cgd.2c01476)
Supplement: Supplementary file 1 — cg2c01476_si_001.pdf [file cg2c01476_si_001.pdf]

Supporting info to:

Does water enable porosity in aluminosilicate zeolites? Porous frameworks versus dense minerals

Karel Asselman,[a] Mohamed Haouas,*[,b] Maarten Houlleberghs,[a] Sambhu Radhakrishnan,[a,c] Wauter Wangermez,[a] Christine E.A. Kirschhock,[a] Eric Breynaert*[,a,c]

[a] *Centre for Surface Chemistry and Catalysis - Characterisation and Application Team (COK-KAT), KU Leuven, Leuven 3001, Belgium*

[b] *Institut Lavoisier de Versailles, Université Paris-Saclay, UVSQ, CNRS, 78000 Versailles, France*

[c] *NMRCoRe – NMR - X-Ray platform for Convergence Research, KU Leuven, Leuven 3001, Belgium*

* Corresponding authors

eric.breynaert@kuleuven.be

mohamed.haouas@uvsq.fr

S1: Supplementary figures and tables

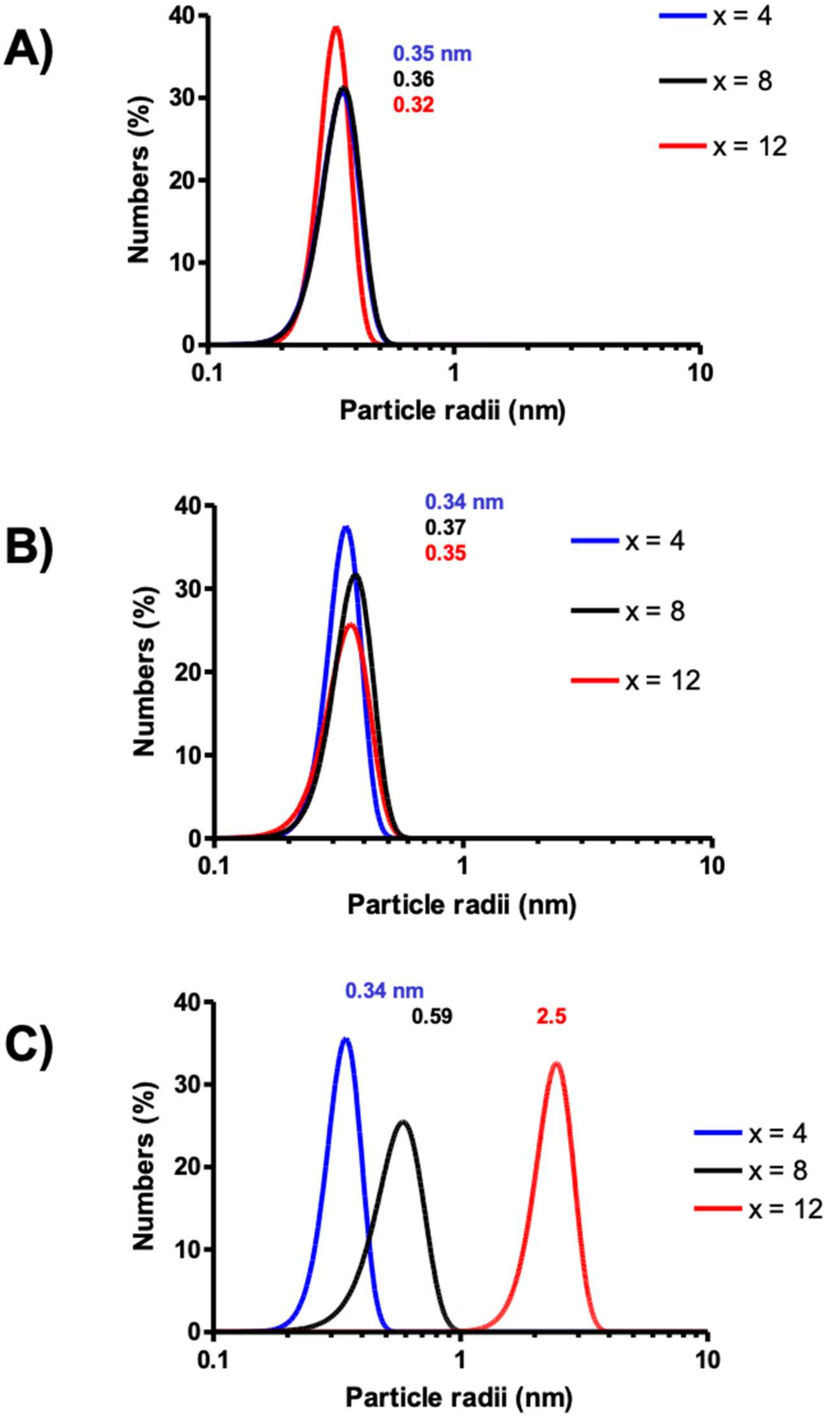

Figure S1: Distribution of particles size in HSILs of compositions 0.5 SiO_2 : 1 KOH : *x* H_2O : a) 0.006, b) 0.013, or c) 0.019 Al_2O_3 as measured by DLS.

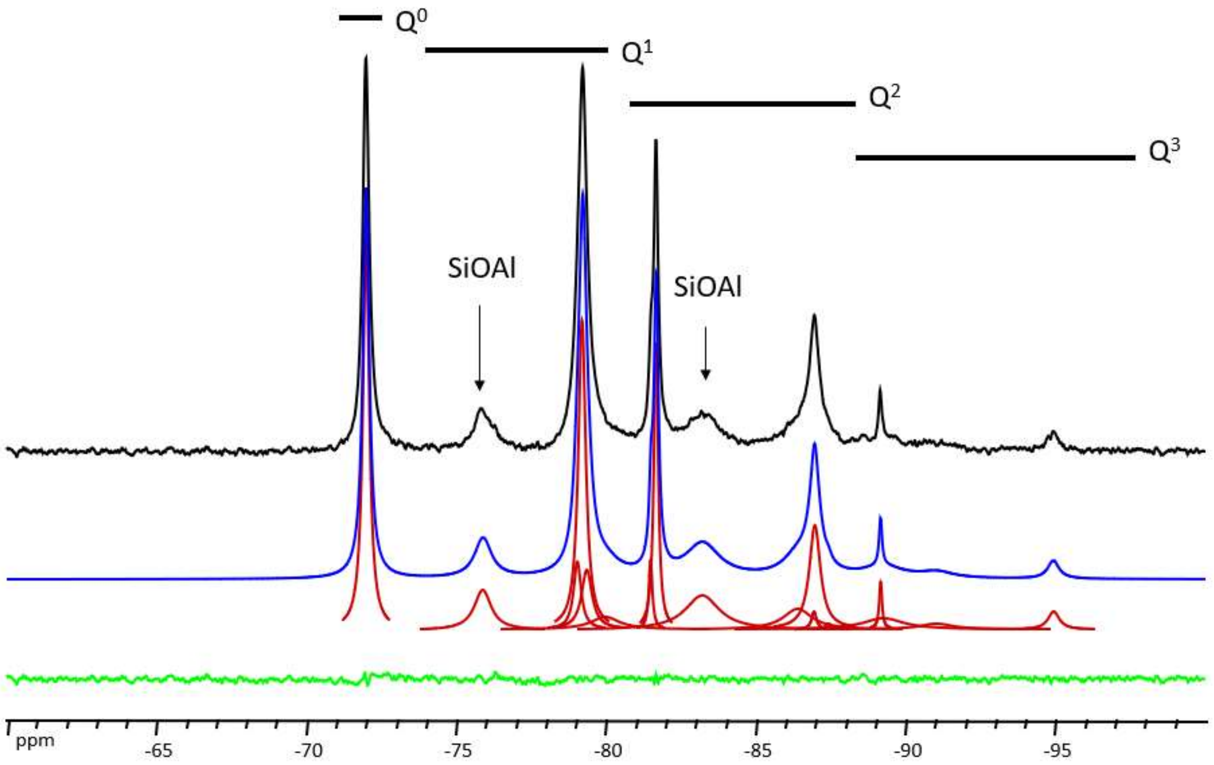

Figure S2. ^{29}Si NMR spectrum in the system 0.5 SiO_2 : 1 KOH : 8 H_2O : 0.019 Al_2O_3. By boosting the aluminate concentration compared to samples discussed in the main article, characteristic resonances for aluminosilicate (SiOAl) oligomers can be discerned. Spectral decomposition allows to determine average Si Q^n connectivity in solution.[1,2] Decompositions of samples A, B and C of the main article are listed in Table S4.

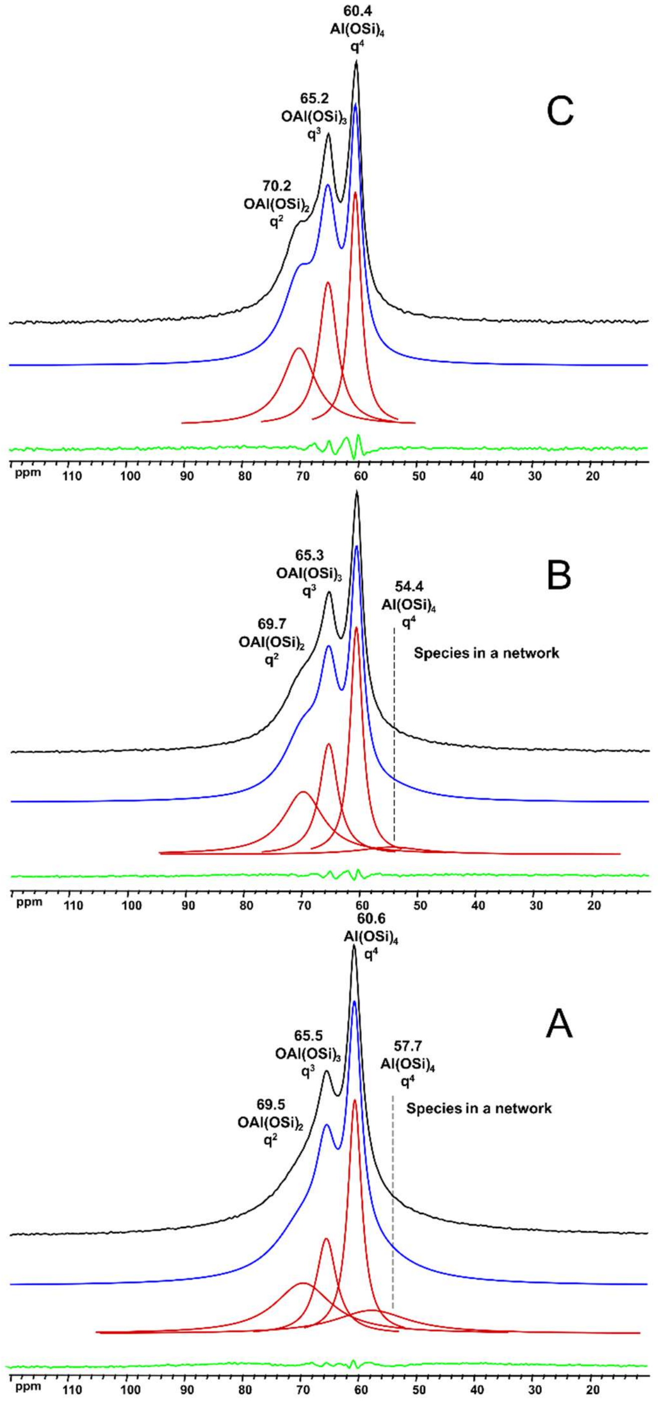

Figure S3. ^{27}Al NMR spectra in the system 0.5 SiO_2 : 1 KOH : *x* H_2O : 0.013 Al_2O_3. *x* = 4 (A), 8 (B), 12 (C). Spectral decomposition allows identification of the three main resonances for q^n species, i.e., $Al(OSi)_n(O)_{4-n}$.

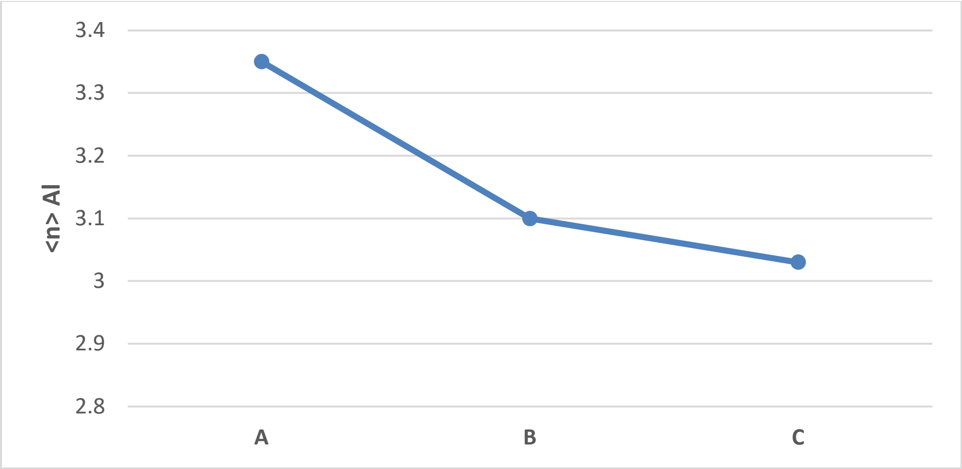

Figure S4: Average connectivity $<n>_{Al}$, derived from decomposition of the ^{27}Al spectra (Figure S3, Table S3).

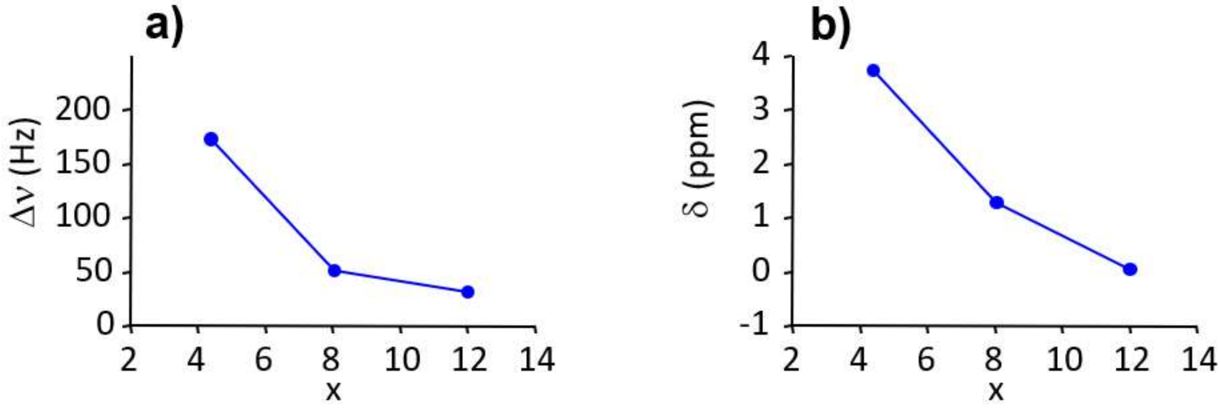

Figure S5. ^{39}K NMR line width Δν and chemical shift δ, as function of water content *x* in 0.5 SiO_2 : 1 KOH : *x* H_2O : 0.013 Al_2O_3. *x* = 4, 8, 12.

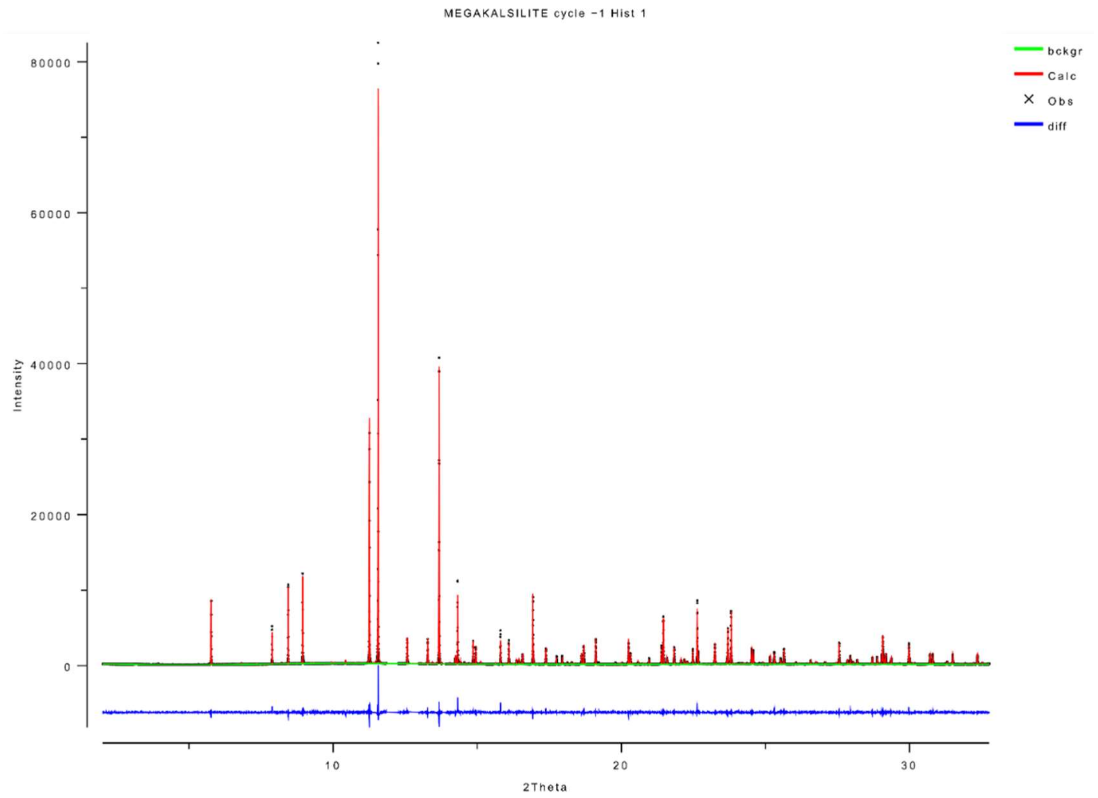

Figure S6: Synchrotron XRD pattern and Rietveld refinement plot of as-made megakalsilite.

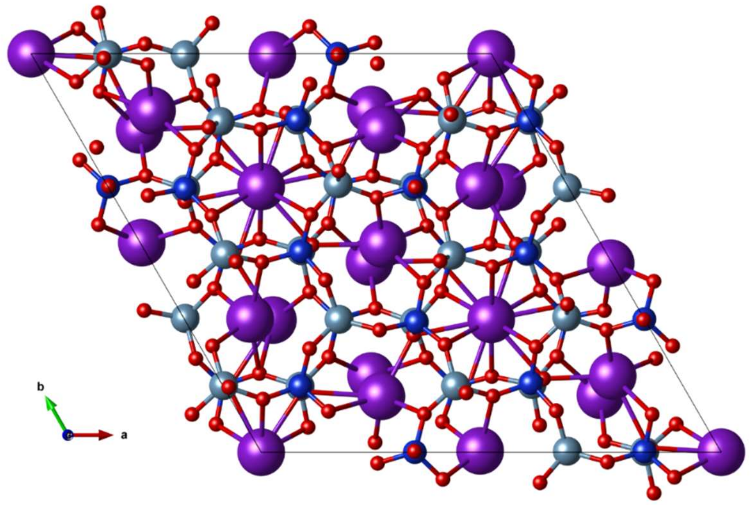

Figure S7: Refined crystal structure of megakalsilite: Silicon (blue), aluminum (grey), oxygen (red), potassium (violet).

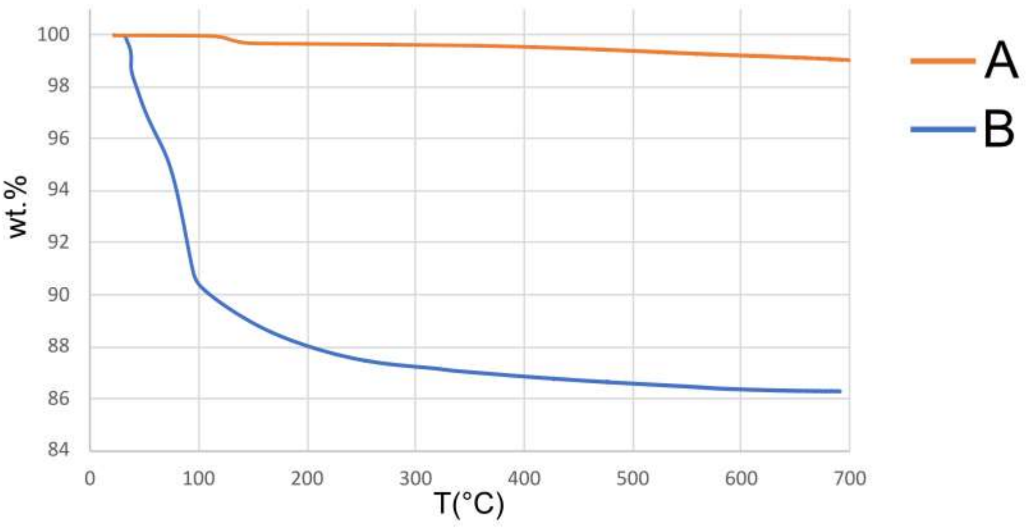

Figure S8: TGA curves of Megakalsilite(A) and MER (B)

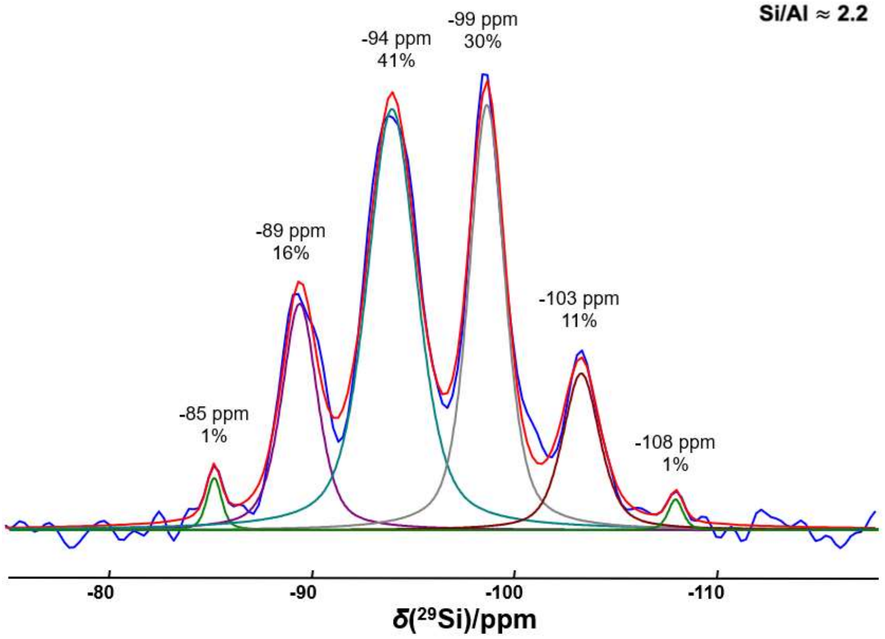

Figure S9: ^{29}Si MAS NMR spectrum and decomposition of sample C (MER).

Table S1: Atomic coordinates of refined megakalsilite structure.

scatterer	x	y	z	U_{iso}
Si1	0.0021(4)	0.1681(5)	0.2086(9)	0.0119
Si2	0.3339(5)	0.3341(6)	0.3125(6)	0.0119
Si3	0.5039(6)	0.1656(5)	0.2009(8)	0.0119
Si4	0.1733(4)	0.5004(6)	0.2030(8)	0.0119
Al1	0.1671(5)	0.1652(6)	0.3209(9)	0.0119
Al2	0.3332(5)	-0.0026(4)	0.2075(6)	0.0119
Al3	0.3393(5)	0.5056(5)	0.3219(10)	0.0119
Al4	0.4961(5)	0.3264(5)	0.3252(9)	0.0119
K1	0	0	0.0212(15)	0.02991
K2	1/3	2/3	0.0193(17)	0.01303
K3	1/3	2/3	0.5224(17)	0.01992
K4	0.00325(34)	0.5257(5)	0.0158(11)	0.01351
K5	0.3343(4)	0.19122(35)	0.0161(14)	0.01756
K6	0.19132(34)	0.3316(4)	0.0141(15)	0.01868
O1	0.0109(6)	0.1632(7)	0.0216(9)	0.0155
O2	0.3346(9)	0.0024(7)	0.0032(7)	0.0155
O3	0.5199(7)	0.1488(7)	0.0194(10)	0.0155
O4	0.1844(6)	0.4782(7)	0.0220(10)	0.0155
O5	0.1331(5)	0.0627(6)	0.2620(14)	0.0155
O6	0.2646(5)	0.5349(6)	0.2863(13)	0.0155
O7	0.5962(5)	0.1901(5)	0.2698(15)	0.0155
O8	0.0949(5)	0.1976(8)	0.2746(10)	0.0155
O9	0.4773(8)	0.2372(7)	0.2152(13)	0.0155
O10	0.1410(8)	0.5668(8)	0.2145(11)	0.0155
O11	0.2598(6)	0.0247(6)	0.2756(16)	0.0155
O12	0.1039(5)	0.4107(6)	0.2809(16)	0.0155
O13	0.4321(5)	0.0764(7)	0.2773(12)	0.0155
O14	0.4288(6)	0.3609(7)	0.2516(16)	0.0155
O15	0.3066(7)	0.4026(6)	0.2551(17)	0.0155
O16	0.2667(5)	0.2391(5)	0.2479(15)	0.0155

*U_{iso} of framework T- and O-atoms were respectively constrained as equal.

Table S2: Rietveld refinement summary of megakalsilite

Wavelength (Å)	0.6223600
Space group	$P6_3$
2θ range (degree)	2.00-33.187
Resolution (Å)	1.09
Number of reflections	705
Lattice constants (Å):	
a	18.099422
b	18.099422
c	8.451138
α	90
β	90
γ	120
Unit cell volume ($Å^3$)	2397.52
Residuals:	
R_{wp}	0.1023
R_{F2}	0.0965

Table S3: Average Al Q^n connectivity <n>, calculated from spectral decomposition of ^{27}Al NMR spectra (Figure S3)

	Al Q^n (%)					
	0	1	2	3	4	<n>
A	0	0	23	19	58	3.35
B	0	0	32	26	42	3.10
C	0	0	32	33	35	3.03

Table S4: Average Si Q^n connectivity <n>, calculated from spectral decomposition of ^{29}Si NMR spectra of Figure 1 in the main text

	Si Q^n (%)					
	0	1	2	3	4	<n>
A	20	36	37	8	0	1.32
B	32	31	32	5	0	1.10
C	44	26	26	3	0	0.89

Table S5: Spectral assignment of the main oligomeric species identified in the ^{29}Si NMR of precursors for samples A, B, and C.[1,2]

Structure	formula	Sample A	Sample B	Sample C
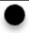	$Si(OH)_4$	-72.0	-72.0	-72.0
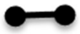	$Si_2O(OH)_6$	-79.2	-79.4	-79.6
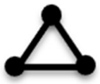	$Si_3O_3(OH)_6$	-81.7	-81.8	-81.8
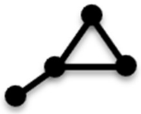	$Si_4O_4(OH)_8$	-78.6<br>-81.5<br>-88.7	-79.2<br>-81.6<br>-88.7	-79.5<br>-81.6<br>-88.9
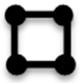	$Si_4O_4(OH)_8$	-89.2	-89.3	-89.4
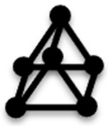	$Si_6O_9(OH)_6$	-89.5	-89.8	-89.8
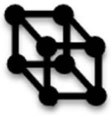	$Si_8O_{12}(OH)_8$	-95.0	-95.3	-95.4

Table S6: ^{29}Si MAS NMR resonances of zeolite W (samples B and C). The observed chemical shifts for the different Si(nAl) environments correspond to those reported by Kennedy et al. for zeolite W (Si/Al = 1.7).[3]

	ppm					
	-107	-103	-98	-94	-89	-85
B	1%	9%	26%	37%	21%	6%
	ppm					
	-108	-103	-99	-94	-89	-85
C	1%	11%	30%	41%	16%	1%

S2: Supplementary discussion

Assignment of impurity phase in megakalsilite (sample A)

While unambiguous assignment from the diffraction is impossible due to minimal observed intensity even in the synchrotron radiation diffractogram, we have strong suspicion that it concerns a potassium hydrogen silicate or a polymorph thereof (KHSiO5).[4,5] From a stoichiometric perspective, they could form from native HSILs without aluminate addition, but have not been observed to do so in our experiments. However, it is possible it co-crystallizes in very small amounts with the megakalsilite. This suspicion is further strengthened by the observation of a weak but very acidic proton signal in the measured ^{1}H-spectrum for this sample (data not shown), which is a signature for these type of alkali silicate phases, due to strong hydrogen bonding between a silanol and deprotonated silanol group.[6]

References

(1) Haouas, M.; Taulelle, F. Revisiting the Identification of Structural Units in Aqueous Silicate Solutions by Two-Dimensional Silicon-29 INADEQUATE. *Journal of Physical Chemistry B* **2006**, *110* (7), 3007–3014. https://doi.org/10.1021/JP0557823.

(2) Goudarzi, N.; Chamjangali, M. A.; Amin, A. H.; Goodarzi, M. Effects of Surfactant and Polyelectrolyte on Distribution of Silicate Species in Alkaline Aqueous Tetraoctylammonium Silicate Solutions Using 29Si NMR Spectroscopy. *Appl Magn Reson* **2013**, *44* (9), 1095–1103. https://doi.org/10.1007/S00723-013-0467-5/FIGURES/7.

(3) Kennedy, G. J.; Afeworki, M.; Hong, S. B. Probing the Non-Random Aluminum Distribution in Zeolite Merlinoite with Ultra-High-Field (18.8 T) 27 Al and 29Si MAS NMR. *Microporous and Mesoporous Materials* **2002**, *52* (1), 55–59. https://doi.org/10.1016/S1387-1811(02)00278-0.

(4) Funk, H.; Stade, H. Die Herstellung Der Kaliumhydrogensilicate (KHSiO3)x, KHSi2O5 (I) Und KHSi2O5 (II) Aus Methanol. *ZAAC - Journal of Inorganic and General Chemistry* **1962**, *315* (1–2), 79–90. https://doi.org/10.1002/zaac.19623150111.

(5) Deng, Z. Q.; Lambert, J. F.; Fripiat, J. J. A Puckered Layered Silicate, KHSi205: Hydrolysis Products and Alkylammonium Intercalated Derivatives. *Chemistry of Materials* **1989**, *1*, 375–380.

(6) Ikeda, T.; Nishide, T.; Nakajima, H.; Kawai, A.; Kiyozumi, Y.; Kodaira, T.; Mizukami, F. Pseudo-Micropores Formed by One-Dimensional Framework with Hydrogen Bonding in CsHSi2O5 Observed by Synchrotron Powder Diffraction and Solid-State MAS NMR. *New Journal of Chemistry* **2008**, *32* (12), 2108–2115. https://doi.org/10.1039/b807879c.
